# Increased CD40 Expression Enhances Early STING-Mediated Type I Interferon Response and Host Survival in a Rodent Malaria Model

**DOI:** 10.1371/journal.ppat.1005930

**Published:** 2016-10-07

**Authors:** Xiangyu Yao, Jian Wu, Meng Lin, Wenxiang Sun, Xiao He, Channe Gowda, Silvia Bolland, Carole A. Long, Rongfu Wang, Xin-zhuan Su

**Affiliations:** 1 Laboratory of Malaria and Vector Research, National Institute of Allergy and Infectious Diseases, National Institutes of Health, Bethesda, Maryland, United States of America; 2 Center for Inflammation and Epigenetics, Houston Methodist Research Institute, Houston, Texas, United States of America; 3 Laboratory of Immunogenetics, National Institute of Allergy and Infectious Diseases, National Institutes of Health, Bethesda, Maryland, United States of America; 4 Department of Biochemistry and Molecular Biology, College of Medicine, Pennsylvania State University, Hershey, Pennsylvania, United States of America; University of Massachusetts Medical School, UNITED STATES

## Abstract

Both type I interferon (IFN-I) and CD40 play a significant role in various infectious diseases, including malaria and autoimmune disorders. CD40 is mostly known to function in adaptive immunity, but previous observations of elevated CD40 levels early after malaria infection of mice led us to investigate its roles in innate IFN-I responses and disease control. Using a *Plasmodium yoelii nigeriensis* N67 and C57BL/6 mouse model, we showed that infected CD40^-/-^ mice had reduced STING and serum IFN-β levels day-2 post infection, higher day-4 parasitemia, and earlier deaths. CD40 could greatly enhance STING-stimulated luciferase signals driven by the IFN-β promoter *in vitro*, which was mediated by increased STING protein levels. The ability of CD40 to influence STING expression was confirmed in CD40^-/-^ mice after malaria infection. Substitutions at CD40 TRAF binding domains significantly decreased the IFN-β signals and STING protein level, which was likely mediated by changes in STING ubiquitination and degradation. Increased levels of CD40, STING, and ISRE driven luciferase signal in RAW Lucia were observed after phagocytosis of N67-infected red blood cells (iRBCs), stimulation with parasite DNA/RNA, or with selected TLR ligands [LPS, poly(I:C), and Pam3CSK4]. The results suggest stimulation of CD40 expression by parasite materials through TLR signaling pathways, which was further confirmed in bone marrow derived dendritic cells/macrophages (BMDCs/BMDMs) and splenic DCs from CD40^-/-^, TLR3^-/-^ TLR4^-/-^, TRIF^-/-^, and MyD88^-/-^ mice after iRBC stimulation or parasite infection. Our data connect several signaling pathways consisting of phagocytosis of iRBCs, recognition of parasite DNA/RNA (possibly GPI) by TLRs, elevated levels of CD40 and STING proteins, increased IFN-I production, and longer host survival time. This study reveals previously unrecognized CD40 function in innate IFN-I responses and protective pathways in infections with malaria strains that induce a strong IFN-I response, which may provide important information for better understanding and management of malaria.

## Author Summary

Malaria parasites infect millions of people and can cause severe disease with diverse symptoms. No highly effective vaccine is available to prevent malaria infection, largely due to the lack of understanding of the molecular mechanisms of host-parasite interactions. Here we investigated the interaction of two important host molecules, CD40 and STING, in host type I interferon (IFN-I) responses to malaria infection using a mouse malaria model. For the first time, we showed that malaria infection could increase CD40 expression, which in turn enhanced the expression of STING, leading to increased production of IFN-I during early infection and better host survival. The increased CD40 expression could be stimulated by phagocytosis of infected red blood cells (iRBCs), parasite DNA/RNA and specific ligands of Toll-like receptors (TLRs), suggesting recognition of parasite materials and signaling by TLRs. This study reveals previously unrecognized protective pathways during malaria infection and provides important information for designing better strategies for malaria control and vaccine development.

## Introduction

Malaria infection triggers strong host immune responses, which may lead to parasite elimination and/or severe pathology [1–5]. Among the immune mechanisms, the role of type I interferon (IFN-I) and interferon-stimulated genes (ISG) in protection against malaria infection has been controversial, but is gaining more attention lately [5, 6]. Recent studies have shown that IFN-I responses involving pathways mediated by cytosolic DNA and RNA sensors/adaptors such as STING (Stimulator of interferon genes), MDA5 (Melanoma differentiation-associated protein 5), and MAVS (Mitochondrial antiviral-signaling protein) can suppress early parasitemia and control liver stage development [7–10], although the mechanism of the IFN-I response in malaria control and pathology is far from clear. STING recognizes AT-rich DNA motifs from malaria parasites [7], whereas MDA5/MAVS can detect parasite RNAs from both sporozoite and blood stages [8–10]. However, whether cyclic-GMP-AMP (cGAMP) synthase (cGAS) plays a role in sensing malaria parasite DNA remains to be determined. STING is an endoplasmic reticulum (ER) adaptor that can activate TBK1 (TANK-binding kinase 1)/IRF3 (IFN regulatory factor 3) pathway to induce expression of IFN-I and ISG genes [11, 12] by directly recognizing dsDNA or cyclic dinucleotides (CDNs) generated by pathogens or metabolized from dsDNA by cGAS [13–15]. STING can also signal through TRAF3 (TNF receptor associated factor 3) or TRAF6 and activate canonical and non-canonical NFκB (Nuclear factor-kappa B) pathways [16].

Another important molecule in host immune response to infections is CD40 (or TNF receptor superfamily member 5, TNFRSF5) that is a receptor expressed on the surfaces of many cell types, including B-cells, monocytes, dendritic cells, endothelial cells, and epithelial cells [17]. CD40 is a type I transmembrane protein with four extracellular cysteine-rich domains that are important for interaction with its ligand CD40L (CD154) on T-cells. The intracellular region of CD40 includes binding domains to TRAF molecules (TRAF1, 2, 3, 5 and 6) [17]. CD40-mediated signal transduction activates multiple pathways, including those signaling through NFκB, STAT3 (Signal transducers and activators of transcription-3), MAPK (Mitogen-activated protein kinase), and other kinases [17–19]. CD40 is mostly known to function in the initiation and progression of cellular and humoral adaptive immunity, including T cell-dependent immunoglobulin class switching, memory B cell development, and germinal center formation [17, 18, 20]. Engagement of CD40 on macrophages and CD40L on T-cells has been shown to promote inflammatory responses in many neurologic diseases [21]. CD40-CD40L interaction between the CD4^+^ T cell and dendritic cells (DCs) primes the DCs to activate CD8^+^ cytotoxic cells [22–24]. The process of CD8^+^ T cell activation is associated with induction of CD70 expression in activated DCs after combined TLR/CD40 stimulations [25]. More recently, ligation of CD40 by CD40L was shown to stimulate IFN-β expression through TRAF2/3 mediated NFκB pathways and sequential de novo synthesis of IRF1 [26, 27]. However, the increase in IFN-β expression by the CD40-mediated NFκB pathway was generally low, compared with those induced by other molecules such as STING or TLR7.

CD40 has been associated with many diseases or disorders [21, 28, 29]. In addition to protection against viral and bacterial infections [30–32], CD40 also contributes to protection against parasitic infections such as *Trypanosoma cruzi*, *Leishmania amazonensis*, and *Plasmodium yoelii* [33–35]. Production of nitric oxide (NO), IL-12, and IFN-γ after ligation of CD40 is critical for controlling *T*. *cruzi* parasitemia [33]. CD40 is required for the maturation of liver dendritic cells, accumulation of CD8^+^ T cells in the liver, and effective APC licensing during *P*. *yoelii* sporozoite infection [34]. Activation and ligation of CD40 and CD40L are also associated with many neurologic and autoimmune diseases that are characterized by elevated levels of IFN-I [28, 29, 36, 37]. Considering the potential role of CD40 in IFN-I response and our observation of up-regulation of IFN-I and CD40 expression in *P*. *yoelii nigeriensis* N67 (N67) infection [8], we investigated the functional roles and the relationship of CD40 and STING in host response to N67 parasite infection and showed that the serum level of IFN-β was significantly reduced in CD40 knockout (KO) mice day 2 after N67 infection, leading to early host death. We further showed that CD40 could greatly enhance STING protein level and STING-mediated IFN-I responses. The effect of CD40 on STING and the IFN-I response was mediated through TRAF2/3 and/or TRAF6 binding domains, leading to changes in STING ubiquitination and protein level. We also showed that various TLR ligands, infected red blood cells (iRBCs), and parasite DNA/RNA could stimulate CD40 expression, establishing a signaling axis of TLR recognition and signaling, increased CD40 and STING levels, elevated IFN-I production, and longer host survival time.

## Results

### CD40 plays a role in IFN-I response, parasitemia control and host survival

C57BL/6 mice infected with N67 parasite induced a strong IFN-I response, including increased expression of genes such as *Cd40*, *Isg15*, *Isg20*, *Ifit2*, *Mx1*, *Mx2*, *Ddx58*, *and Usp18* genes (**S1A and S1B Fig**), leading to suppression of N67 parasitemia [8, 38]. Ligation of CD40 with CD40L was recently shown to activate the NFκB pathway resulting in enhanced IFN-I response in carcinoma cells [27]. The elevated levels of *Cd40* transcripts in the N67 infected mice suggest that it may play a role in regulating host IFN-I response to malaria infection.

**Fig 1:**
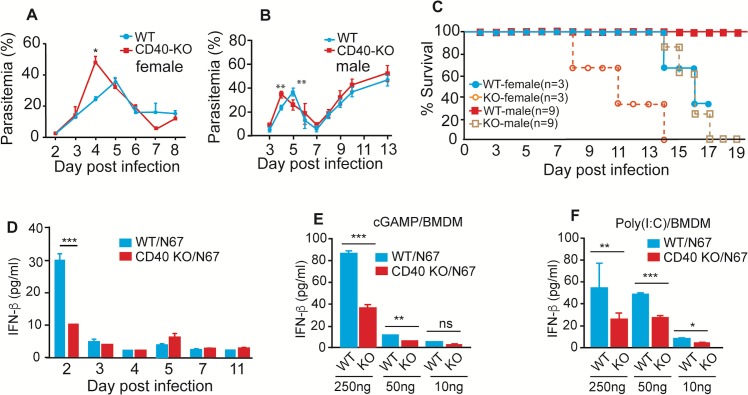
CD40 KO significantly affects day 4 parasitemia, host mortality, IFN-β response *in vivo* and *in vitro*. (**A** and **B**) Daily parasitemia of N67 infected female (**A**) or male (**B**) C57BL/6 mice with or without *Cd40* gene. (**C**) Host mortality after infection with N67 parasite. (**D**) Serum levels of IFN-β in mice with or without *Cd4*0 gene after infection with N67 parasites. **(E)** Significantly lower IFN-β levels in culture supernatants of bone marrow-derived macrophage (BMDM) cells without *Cd40* gene after stimulation with cGAMP. Cells were stimulated for 8 h before ELISA measurement. (**F**) The same experiments as in (**E)**, but stimulated with poly(I:C). Experiments in D were done in female mice, and those in E-F were cells from female mice. All data are means+s.d. from three experiments or at least three mice (**A-C**); *t*-test, *P<0.05; **P<0.01; ***P<0.001.

To investigate the role of CD40 in IFN-I mediated protection against malaria infection, we infected wild type (WT) and CD40^-/-^ mice with N67 and monitored parasitemia and host mortality. Compared with WT mice, both infected male (n = 9) and female (n = 3) CD40^-/-^ mice had significantly higher day 4 parasitemia (*P* = 0.0078 for male; *P* = 0.0137 for female) (**Fig 1A and 1B**) and died earlier (Ave survival days, WT over 25 days vs KO 15 days for male, *P*<0.001; 15 days vs 10 days for female, *P* = 0.0623) than the WT mice (**Fig 1C**). As expected, the day 2 (24 h after parasite injection) serum levels of IFN-β were significantly lower in the CD40^-/-^ mice than those of WT mice after parasite infection; however, the IFN-β level quickly returned to low levels despite continuous increase of parasitemia (**Fig 1D**). These observations were similar to our previous measurements of IFN-I in splenic cDCs after N67 infection [8]. These results suggest that CD40 played a role, despite not a major role, in the IFN-β response and in controlling early parasitemia during N67 parasite infection, e.g., lower day 2 IFN-β level in the CD40^-/-^ mice resulted in higher day 4 parasitemia and earlier host death. The decline of IFN-β after day 2 also suggests active regulation of IFN-I levels during the infection.

To further investigate the effects of CD40 on IFN-I response *in vitro*, we obtained bone marrow derived macrophages (BMDM) from WT and CD40^-/-^ mice, stimulated the cells with cGAMP or poly(I:C) *in vitro* and measured IFN-β protein levels in the culture media. Significant reduction of IFN-β protein was observed in CD40^-/-^ BMDM relative to those from WT mice when stimulated with 250 ng or 50 ng cGAMP, respectively (**Fig 1E**). Similarly, significantly lower levels of IFN-β were detected in the cells from the CD40^-/-^ mice when stimulated with poly(I:C) (**Fig 1F)**. These results show that CD40 plays a role in cGAMP and poly(I:C) stimulated IFN-β responses, likely mediated by STING/MAVS/TLR mediated pathways.

### CD40 enhances STING/MAVS mediated IFN-I response

**Fig 2:**
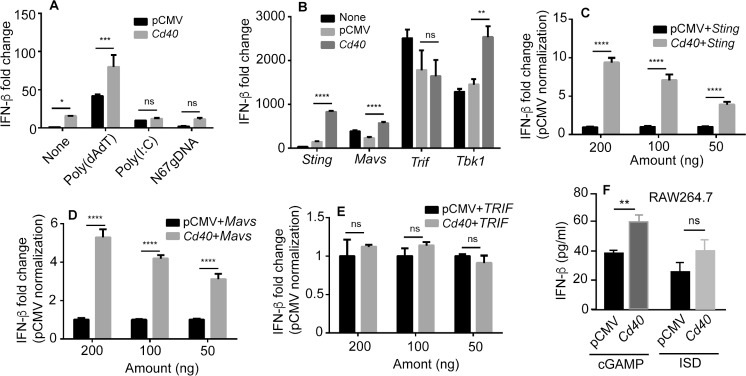
CD40 enhances IFN-β expression through STING/MAVS mediated pathways. (**A**) IFN-β level, measured as luciferase signals driven by IFN-β promoter, in 293T cells after transfection of a pCMV plasmid containing the gene encoding CD40 and stimulated with the indicated agents. Luciferase activities were measured 18 h after transfection of 200 ng plasmid each. (**B**) Co-transfection of 293T cells with plasmids containing the *cd40* and the indicated genes. IFN-β level, measured as luciferase signals driven by IFN-β promoter in 293T cells, were measured as in (**A)**. (**C-E**) Dose-response assays as done in **(B)**. (**F**) IFN-β protein levels in culture supernatants of RAW Lucia cells measured using ELISA after stimulation with cGAMP or interferon stimulatory DNA (ISD) for 6 h; both are STING ligands. All data are means+s.d. from three experiments; *t*-test, *P<0.05; **P<0.01; ***P<0.001.

To investigate the mechanism as to how CD40 regulates IFN-I response, we transfected 293T cells with plasmids containing genes encoding CD40, a luciferase reporter plasmid driven by the IFN-β promoter (IFN-β-luciferase), and a plasmid containing renilla luciferase as control and measured luciferase activities as described previously [8]. Compared with cells transfected with control pCMV plasmid vector, introduction of the plasmid containing CD40 gene significantly increased luciferase signals with or without poly(dAdT) stimulation 18 h after transfection (**Fig 2A**). However, the increases in luciferase signals were not significant when stimulated with poly(I:C) or N67 parasite gDNA (**Fig 2A**). We next co-transfected the cells with plasmids containing CD40 plus those carrying genes encoding STING, MAVS, TRIF, or TBK1 and showed that co-transfection with genes encoding STING, MAVS or TBK1 could significantly increase luciferase signals, but not those encoding TRIF (**Fig 2B**). The effects of CD40 on enhancing the STING and MAVS activities were further confirmed in dose-response experiments (**Fig 2C and 2D**). Consistently, no differences in dose-response were observed when the cells were co-transfected with genes encoding CD40 and TRIF (**Fig 2E**). Similar effects of CD40 on the STING pathway were observed in RAW Lucia cells; significantly higher IFN-β protein level was observed when RAW Lucia cells were transfected with a plasmid containing CD40 gene and then stimulated with cGAMP (for ISD, *P* = 0.052) that targets the STING pathway (**Fig 2F**).

### CD40 and STING affect each other’s expression *in vitro* and *in vivo*

**Fig 3:**
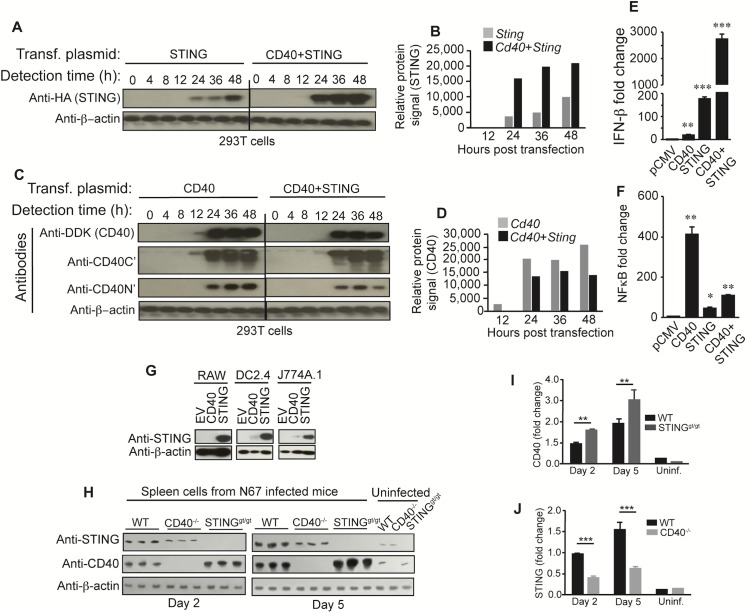
Interaction and co-regulation of CD40 and STING expression. (**A** and **B**) Western blots showing dynamics of STING protein levels (**A**) and quantifications of the protein bands (**B**). Cells (2 × 10^5^) were transfected with plasmids carrying genes encoding CD40 and/or STING. Protein extracts collected at different time points were detected using antibodies against HA (STING). (**C** and **D**) Western blots showing dynamics of CD40 protein levels (**C**) and quantifications of the protein bands (**D**). Three antibodies were used to detect CD40 protein; anti-DDK tag, anti-CD40 C-terminus and N-terminus, respectively. (**E**) Stimulation of STING mediated IFN-β response by CD40. Cells (2 × 10^5^) were transfected with plasmids containing the indicated genes, and luciferase signals driven by IFN- β promoter were measured 24 h post transfection. (**F**) The same experiments as (**E)**, except measuring luciferase signals driven by NFκB promoter (signaling of CD40). For (**E**) and (**F**), data are means+s.d. from three experiments; *t*-test, *P<0.05; **P<0.01; ***P<0.001. **(G)** CD40 increased STING level in DC2.4 and J774A.1 cell lines. Cells (2 × 10^5^) were electroporated with empty plasmid (EV) or plasmids containing gene encoding CD40 or STING, and proteins were detected using anti-STING antibody. Anti-β-actin was used as loading control. **(H)** CD40 and STING expression in splenic tissues of wild type (WT), CD40^-/-^ or STING^gt/gt^ mice (3 each) after N67 infection. Anti-CD40 or anti-STING antibodies were used to detect protein expression in tissue homogenates of uninfected, day 2 or day 5 infected mice. Anti-β-actin was used as loading control. (**I-J**) Signal intensities from the protein bands in (**H**). For the day 2 and day 5 infected groups, the bars are means+s.d. from three mice; for the uninfected (Uninf.), the values are from single mouse. **P<0.01; *** *P*<0.001.

The effect of CD40 on enhancing IFN-I production could be mediated by direct stimulation of STING expression and/or through protein modifications. To investigate the mechanisms of how CD40 enhances STING-mediated IFN-I production, we determined protein expression at different time points after introduction of the CD40 and/or STING plasmids into 293T cells and found that the levels of STING expression were greatly elevated 24–48 h post co-transfection with the CD40 expression vector, with the highest STING level at 48 h (**Fig 3A and 3B**).

We also used various antibodies to detect CD40 protein expression. Protein bands could be detected from 12–48 h using antibodies against DDK-tag fused to CD40 and against N’- or C’-termini of CD40 (a small band could be seen 12 h post infection using anti-C-terminus and anti DDK tag). Interestingly, the presence of STING decreased CD40 levels between 12–48 h (**Fig 3C and 3D**). The decrease in CD40 protein appeared to be due to cleavage of CD40 N’-terminus because of the presence of lower molecular weight bands and the reduction in protein signals detected by anti-C’- and N’-terminal antibodies, respectively (**Fig 3C**). The higher STING protein levels in cells co-transfected with CD40 likely contributed to the elevated luciferase signals (**Fig 3E**). Similarly, the CD40 protein degradation when co-transfected with STING could also explain the reduced NFκB signaling in cells co-transfected with STING (**Fig 3F**). Both STING and CD40 have been shown to activate the canonical and non-canonical NFκB pathways and IFN-I production by recruiting various TRAF molecules [16, 27]. However, STING is a stronger IFN-I inducer than CD40, whereas CD40 is better at activating the NFκB pathway (**Fig 3E and 3F)**. These results show that CD40 can greatly enhance the STING-mediated IFN-I response, whereas STING can inhibit the CD40-mediated NFκB pathway.

We also investigated the effects of CD40 on endogenous STING expression in different cell lines and in mice after infection with N67 parasite. Introduction of CD40 expressing plasmid could increase STING levels in DC2.4 and J774A.1 cells, but not obvious in the RAW Lucia cells (**Fig 3G**). In the spleen of uninfected mice, both CD40 and STING were expressed at low levels; however, both protein levels were increased in the spleen of N67-infected WT mice day-2 and -5 *p*.*i*. (**Fig 3H–3J and S2 Fig**). Compared with WT mice, STING expression was significantly reduced in infected CD40^-/-^ mice on both days, whereas CD40 levels were significantly increased in the infected STING^gt/gt^ mice (**Fig 3H–3J**). We also investigated CD40 and STING expression in TRIF^-/-^, TLR3^-/-^, TLR4^-/-^, and MyD88^-/-^ mice infected with N67 and showed that CD40 protein level was decreased in all the KO mice, (**S2A and S2B Fig**), although the reductions in STING in the TLR3^-/-^, TLR4^-/-^, and MyD88^-/-^ mice were minimum. These results suggest that TRIF can positively regulate CD40 expression *in vivo*, whereas STING negatively affects CD40 protein level in some cell lines (dendritic cells and monocytes) and *in vivo* during malaria infection.

### CD40 signals through TRAF binding domains to regulate STING and IFN-I expressions

**Fig 4:**
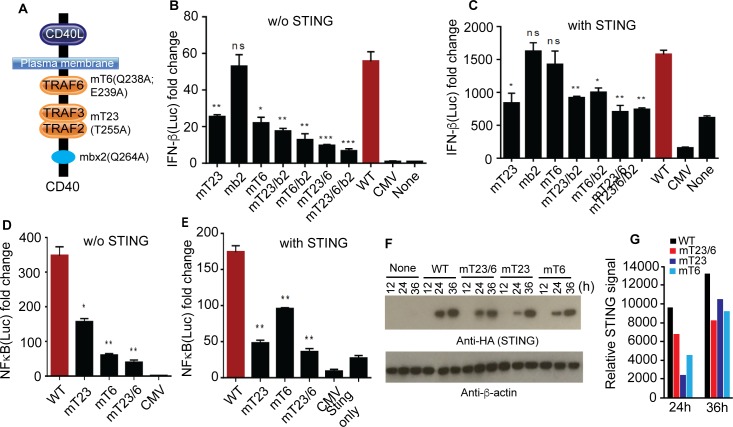
Mutations in CD40 TRAF binding domains affect STING mediated IFN-β response. (**A**) Diagram of CD40 showing various amino acid substitutions in the TRAF binding domains. For example, mT6 (Q238A, E239A) stands for mutations in TRAF 6 binding domain with amino acid substitution at Q238A and E239A. Mbx2 is the box2 at the C-terminal of CD40 as described [17]. (**B**) Luciferase signals driven by IFN-β promoter from 293T cells (2 × 10^5^) transfected with plasmids carrying wild type (WT) or different *Cd40* mutant genes were measured 24 h after lipofectamine transfection. Mutations in one or more TRAF binding domains/box are as indicated. (**C**) The same experiments as in (**B**) with co-transfection of *Sting* gene. (**D)** Luciferase signals derived by NFκB promoter from 293T cells transfected with plasmids carrying WT or mutations in TRAF23 or TRAF6 domains of CD40. (**E**) The same experiments as (**D)** but co-transfected with *Sting* gene. (**F**) STING protein expression levels after co-transfection of 293T cells (2 × 10^5^) with WT *Cd40* or *Cd40* with mutations in the TRAF binding domains for 24 h, and cell lysates were detected on Western blot. (**G**) Quantitative measurements of protein levels in (**F)**. For (**B-E**), data are means+s.d. from three experiments; *t*-test, *P<0.05; **P<0.01; ***P<0.001.

CD40 has intracellular domains that are known to bind TRAF2, TRAF3, and TRAF6 in canonical or non-canonical NFκB pathways [18]. Recently, the TRAF2 and TRAF3 binding domains of CD40 were also found to be responsible for IFN-β gene transactivation through NFκB pathways [27]. Similarly, STING can also function by signaling through TRAF3 and TRAF6 to modulate canonical and non-canonical dsDNA-mediated NFκB activation [16]. To investigate the functional role of CD40 TRAF domains in the IFN-I signaling, we first introduced amino acid substitutions in the CD40 TRAF2/3 and TRAF6 binding domains based on known mutations that are critical for its binding to TRAF molecules (**Fig 4A**) [16, 17, 39]. Plasmids containing one or more amino acid substitutions in the TRAF binding domains were generated using a site mutagenesis kit (Quick Change II, Agilent Technologies) and the expected substitutions were confirmed after DNA sequencing of the mutagenized clones (**S3 Fig**). Substitutions in the binding domains of TRAF2/3 (T255A), TRAF6 (Q238A; E239A), TRAF2/3/box2 (T255A; Q264A), TRAF6/box2 (Q238A; E239A; Q264A), TRAF2/3/6 (T255A; Q238A; E239A), and TRAF2/3/6/box2 (T255A, Q238A, E239A, and Q264A) significantly reduced luciferase signals driven by the IFN-I promoter in 293T cells, whereas the mutation in box2 (Q264A) alone did not alter the luciferase signals (**Fig 4A and 4B**). Generally, simultaneous mutations affecting two TRAF domains reduced luciferase signals more significantly than those affecting one single TRAF domain or box. These results show that TRAF2/3 and TRAF6 binding domains play a role in stimulating STING-mediated IFN-I response.

The luciferase signals were much higher when CD40 and STING were co-transfected into the 293T cells compared with expression of CD40 alone (**Fig 3E**), suggesting that the impact of CD40 on IFN-I production is more significant when acting as regulator of STING than signaling through its own NFκB pathways. At the presence of a plasmid encoding STING, only the T255A mutation affecting TRAF2/3 could significantly reduce the luciferase signal, although all the constructs with mutations in more than one domain also had significant effects (**Fig 4C**). The results again suggest that TRAF2/3 binding domain is the key domain affecting CD40 enhancement of the STING-mediated IFN-I response. As previously reported [18], mutations in the TRAF2/3 and TRAF6 domains also significantly affect NFκB activation with or without STING expression, although the effect of TRAF6 mutations on NFκB activation was less than that of TRAF2/3 mutation in the presence of STING (**Fig 4D and 4E**). Mutations in the TRAF binding domains could indeed affect the ability of CD40 to enhance STING protein levels (**Fig 4F and 4G**). These results suggest that the stimulation of STING expression and therefore IFN-I response is mainly mediated through the TRAF binding domains of CD40.

We also investigated whether overexpression of TRAF2, TRAF3, or TRAF6 affected

IFN-β and NFκB signaling with or without the presence of CD40, STING or both. As expected, overexpression of TRAF3, but not TRAF2 or TRAF6, greatly suppressed CD40 mediated NFκB activation (**S4A–S4C Fig**), suggesting inhibition of NIK disassociation from cIAP1/2 and of NFκB activation [40, 41]. The patterns of TRAF2 and TRAF3 in affecting IFN-β responses were similar; both enhanced IFN-β signal when transfected with STING alone, but decreased IFN-I production when co-transfected with CD40 and STING (**S4D** and **S4E Fig**). TRAF6 increased IFN-β signals in all situations (**S4F Fig**). These results suggest that TRAF2/3 and TRAF6 play a role in the CD40 and/or STING signaling pathways leading to IFN-I production, but they may have different roles at high levels of CD40 and STING; TRAF2 and TRAF3 could affect the degradation of CD40, STING, or proteins in pathways leading to increased STING level or other IFN-I signaling.

### CD40 interacts with STING through TRAFs and reduces STING ubiquitination

**Fig 5:**
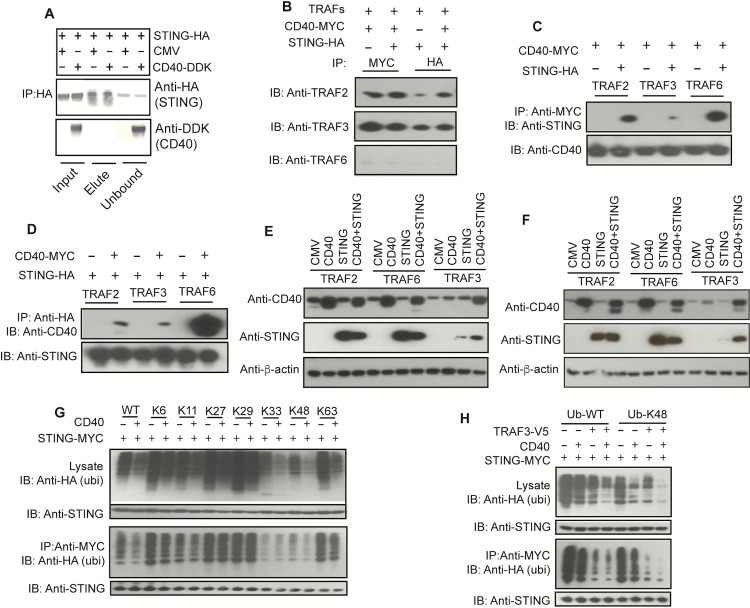
CD40 modulates STING ubiquitination and degradation through interaction with TRAFs. (**A**) Co-immunoprecipitation (co-IP) of CD40 and STING. Co-IP of CD40 and STING 24 h after transfecting genes encoding CD40 and STING. Anti-HA antibody was used to pull-down protein complex in 293T cells transfected with DDK-tagged CD40, and proteins were detected using anti-HA (STING) or anti-DDK (CD40). No direct interaction of CD40 and STING was detected. (**B**) Co-IP using anti-Myc to pull-down CD40 and anti-HA to pull-down STING and immunoblot (IB) detection of TRAF molecules after co-transfection with TRAF2, TRAF3, or TRAF6, respectively. (**C** and **D**) The same experiments as in (**B**), but pulling-down using anti-Myc (CD40) and detection using anti-HA (STING) (**C**), or vice versa (**D**). (**E**) Effects of over expression of TRAF2, TRAF3, or TRAF6 on CD40 and STING protein levels 24 h after co-transfection of plasmids with indicated genes. (**F**) The same experiments as (**E**) but 48 h after co-transfection. (**G**) Detection of protein ubiquitination after co-transfection of plasmids encoding HA-tagged wild type or mutant ubiquitins with specific lysine (K) sites and with or without those encoding CD40 and STING. Anti-Myc was used to detect STING. (**H**) The same experiments as in (**G**), except Myc tagged STING was pulled-down before detection of HA-tagged ubiquitins. All the assays were performed in 293T cells (2 × 10^5^). IB, immunoblotting; IP, immunoprecipitation (pulled-down).

Because CD40 and STING regulate each other’s protein levels and molecular signaling, we next investigated whether there was any physical interaction between the two molecules by performing co-immunoprecipitation (co-IP) experiments, although CD40 and STING are known to localize at the plasma membrane and ER, respectively [12, 17]. Protein complexes were pulled down using anti-HA antibody after co-transfection of 293T cells with plasmids containing HA-tagged STING and/or DDK/Myc-tagged CD40, and the bound/unbound proteins were detected using anti-DDK (or anti-CD40) and anti-HA antibodies. CD40-DDK was detected in the unbound fraction, but not in the fraction pulled down by anti-HA antibody (**Fig 5A**). As expected, these results suggest that CD40 does not bind to STING directly. The lack of direct interaction is consistent with IFA results showing lack of co-localization of the two proteins in both 293T cells (**S5A Fig)** and in Hela cells **(S5B Fig**).

Next we investigated how TRAFs interact and regulate CD40 and STING protein interaction. We transfected 293T cells with V5-tagged TRAF2, TRAF3, or TRAF6 and CD40-DDK/Myc, STING-HA, or both, used anti-Myc or anti-HA to pull-down protein complexes, and detected the pulled-down proteins using anti-TRAFs (V5), anti-STING (HA), or anti-CD40 (Myc), respectively. We found that both CD40 and STING were directly associated with TRAF3, TRAF2, and TRAF6, and CD40 and STING could be pulled down together, at least with overexpression of the TRAFs (**Fig 5B–5D**), suggesting the presence of CD40/TRAF/STING complex. Compared with TRAF2 and TRAF6, overexpression of TRAF3 decreased CD40 protein level at 24 h (**Fig 5E**) and 48 h (**Fig 5F**) post transfection. Similarly, overexpression of TRAF3 greatly degraded STING, and co-expression of CD40 could protect some STING from degradation (**Fig 5E and 5F**). These observations can be explained by competition for binding the TRAFs between CD40 and STING. Increased binding of TRAF3 to CD40 decreased its availability to STING, leading to reduced STING degradation.

CD40 stimulation has been shown to facilitate K48-linked polyubiquitination and degradation of TRAF3, causing disassociation of NIK from cIAP1/2p100 processing and NFκB activation [40]. To investigate the mechanism of how increased CD40 expression affects STING protein levels, we assayed for STING ubiquitination with or without overexpression of CD40 in 293T cells. Our results showed that addition of CD40 could reduce STING ubiquitination, including at position K48 and K63 (**Fig 5G and 5H**). The TRAF molecules, particularly TRAF2 and TRAF3, could play an important role in STING ubiquitination and degradation.

### TLR ligands and iRBCs can stimulate CD40 and STING expression and increase ISRE-luc signal

**Fig 6:**
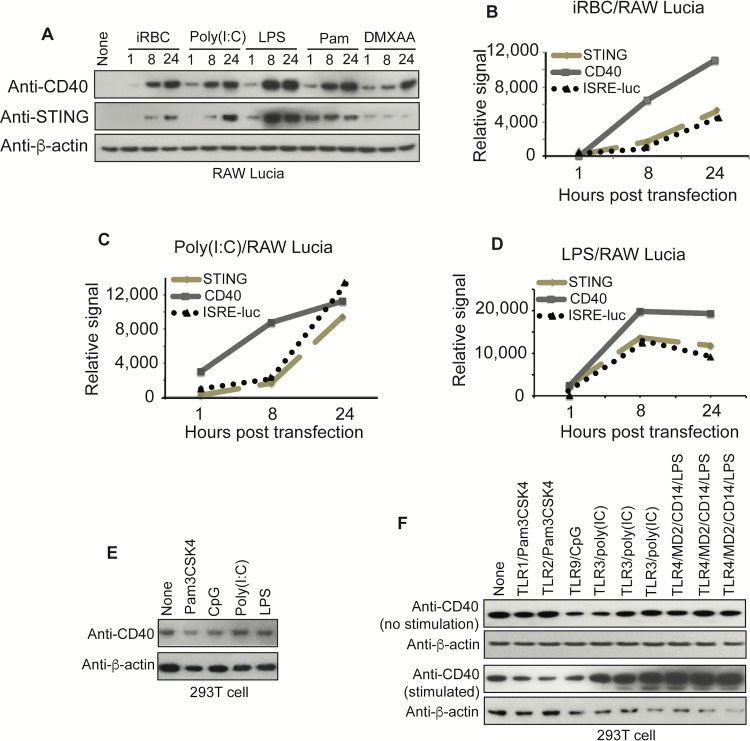
Stimulation of CD40 and STING expression and ISRE driven luciferase activities by TLR ligands and infected red blood cells (iRBCs). (**A**) RAW Lucia cells (2 × 10^5^) were stimulated with different ligands for indicated times, and protein levels of CD40 and STING were detected using an antibody against C-terminus of CD40 and anti-STING, respectively. Pam is Pam3CSK4. (**B**) Plots of protein band intensities of CD40 and STING and ISRE-luciferase signals 1, 8, and 24 h after infected red blood cell (iRBC) stimulation. All data for ISRE-luciferase signals are averaged values from three experiments. (**C**) The same plots as in (**B**) after infected poly(I:C) stimulation. (**D**) The same plots as in (**B**) except stimulation with LPS. (**E**) Western blot detection of CD40 protein levels in 293T cells (2 × 10^5^) after TLR ligand stimulations for 12 h. (**F**) Expression of CD40 in 293T cells (2 × 10^5^) transfected with various TLRs for 18 h and with or without ligand stimulations for another 12 h before harvesting for proteins and Western blot analysis. The three TLR3 and TLR4 experiments, respectively, were conducted using plasmids containing the genes from different sources.

Many ligands are known to stimulate CD40 expression; CD40L, TNF-α, type I and II interferons, and LPS are known to induce or modulate CD40 expression [21, 42]. LPS was shown to induce IFN-β that in turn stimulated CD40 expression through TRAM/TRIF/IRF3 signaling [42]. To investigate possible mechanisms that stimulate CD40 expression and IFN-I production during malaria infection, we stimulated RAW Lucia cells with iRBCs, poly(I:C) (TLR3 ligand), LPS (TLR4 ligand), Pam3CSK4 (TLR1/2 ligand), and DMXAA (mouse STING ligand) and detected CD40 and STING expressions on Western blot 1, 8, and 24 h post stimulation. In all cases, higher CD40 expression levels were observed with increased incubation time; for STING, increased protein levels over time were observed only in cells stimulated with iRBCs and poly(I:C) (**Fig 6A**). We also measured luciferase activities driven by ISRE promoter and plotted the luciferase signals against CD40 and STING protein band intensities after stimulation of RAW Lucia cells for 1, 8, and 24 h. Interestingly, there were similar trends of protein intensities of CD40 and STING and luciferase signals after stimulations of the cells with poly(I:C), LPS, and iRBCs (**Fig 6B–6D**), suggesting direct relationships between CD40/STING levels and IFN-I response, but not those stimulated with Pam3CSK4 and DMXAA (**S6A** and **S6B Fig**). We next stimulated the RAW Lucia cells with additional ligands for 24 h and measured luciferase signals driven by ISRE promoter and CD40 protein expression with or without lipofectamine treatment on Western blot (**S6C** and **S6D Fig**). Compared with un-stimulated cells, iRBCs, DMXAA, cGAMP, poly(I:C), LPS, and Pam3CSK4 again induced higher ISRE luciferase signals without lipofectamine, suggesting that TLR1/2, TLR3, and TLR4 may play a role in enhancing CD40 levels. However, the higher luciferase signals induced by poly(dAdT) and cGAMP at the presence of lipofectamine indicated that some of the signals could be from mechanisms bypassing signaling by receptors on the cell surface. CD40L, IFN-γ or both did not induce significant amounts of luciferase signals or CD40 protein expression. Stimulation of 293T cells with CD40L did not increase CD40 protein level either (**S6E Fig**).

To confirm that TLRs play a role in CD40 expression, we transfected 293T cells with TLR1, TLR2, TLR3, TLR4 and TLR9 for 18 h and then detected CD40 expression with or without TLR ligand [Pam3CSK4, CpG, poly(I:C), and LPS] stimulation for another 12 h. Incubation with individual TLR ligands alone or transfection of TLRs into the 293T without ligand stimulation did not increase CD40 levels (**Fig 6E and 6F**); however, the presence of TLR3 and TLR4 plus their ligands, respectively, greatly increased CD40 expression (**Fig 6F**). These results further confirm the role of TLR3 and TLR4 in stimulation of CD40 expression.

### Phagocytosis of iRBCs and parasite DNA/RNA stimulate CD40 expression and ISRE induced signal

**Fig 7:**
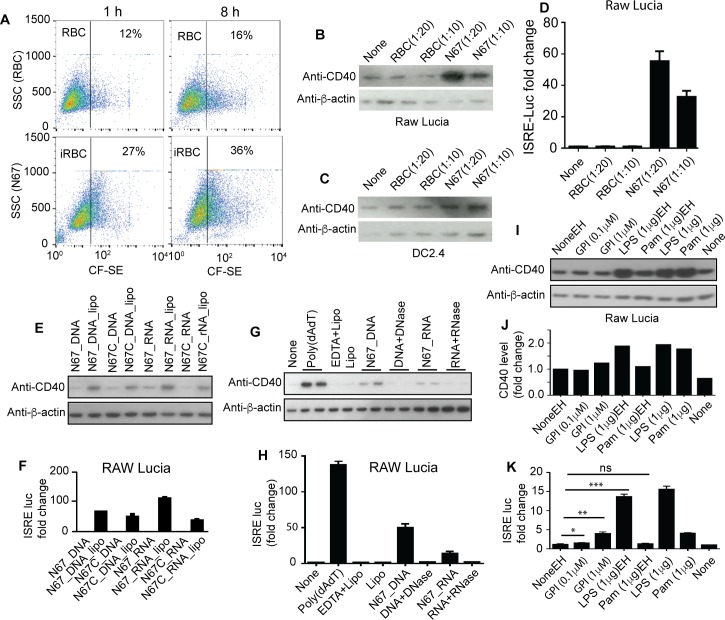
Stimulation of CD40 expression and ISRE driven luciferase signals by infected red blood cells (iRBCs), parasite DNA/RNA, or parasite GPI. (**A**) Flow cytometry counts of RAW Lucia cells containing N67 iRBCs 1 h and 8 h post incubation. The top two panels are RBCs, and the bottom panels are iRBCs. SSC, side-scattered light; CF-SE, signals of carboxyfluorescein succinimidyl ester (CF-SE) labeled RBCs or iRBCs (**B**) CD40 protein expression in RAW Lucia cells after incubation with RBCs or iRBCs. The antibody was against an epitope at C-terminus of the CD40 protein. Anti-β-actin was used as protein loading control. (**C**) The same as (**B**) but in DC2.4 cells. (**D**) ISRE promoter driven luciferase signals in RAW Lucia cells after ingestion with RBCs or iRBCs. (**E**) CD40 protein expression in RAW Lucia cells stimulated with parasite genomic DNA or RNA with or without the presence of lipofectamine. (**F**) Luciferase activities driven by ISRE promoter after stimulation with parasite DNA or RNA as in (**E**). (**G**) CD40 protein expression in RAW Lucia cells stimulated with parasite DNA or RNA with or without DNase or RNase treatment, respectively. EDTA and/or lipofectamine (Lipo) were used as buffer controls. (**H**) Luciferase activities driven by ISRE promoter after stimulation with parasite DNA or RNA with or without DNase or RNase treatment, respectively, as in (**G**). (**I**) CD40 protein expression in RAW Lucia cells stimulated with LPS, Pam3CSK4, and *Plasmodium falciparum* GPI (glycophosphatidylinositol) dissolved in ethanol (marked with EH) or H_2_O. (**J**) Protein band intensities in (**I**) relative to non-stimulated in ethanol (NoneEH = 1). (**K**) Luciferase activities driven by ISRE promoter in RAW Lucia cells stimulated with LPS, Pam3CSK4, and *P*. *falciparum* GPI as in (**I**). For (**D**), (**F**), (**H**), and (**K**), data are means+s.d. from three experiments; *t*-test, *P<0.05; **P<0.01; ***P<0.001. For all the experiments, 5 × 10^5^ cells were used.

To investigate parasite ligands that may play a role in this process, we assayed various parasite materials for stimulation of CD40 expression and IFN-I responses. Because malaria parasites reside within RBCs, one potential mechanism for the host cells to detect parasite molecules is through phagocytosis and digestion of iRBCs. We therefore incubated iRBCs (schizonts) with RAW Lucia cells and measured CD40 levels and luciferase activities driven by the ISRE promoter. More than twice the numbers of iRBCs were phagocytized than those of uninfected RBCs (**Fig 7A**) and higher amounts of CD40 protein were detected 8 h post co-incubation in RAW Lucia cells (**Fig 7B)** and slightly higher amounts in DC2.4 cells **(Fig 7C)**. The higher level of CD40 level also led to increased luciferase signals driven by ISRE promoter (**Fig 7D**), although the luciferase signals could also include signals from other IFN-I signaling pathways. Phagocytosis of RBCs did not induce CD40 expression or luciferase signal (**Fig 7B and 7D**). The lack of CD40 expression and luciferase signal after phagocytosis of RBCs and the dose-dependent signals suggest that the CD40 expression and luciferase signals detected were parasite specific.

We next used parasite DNA and RNA to stimulate CD40 and ISRE luciferase expression in RAW Lucia cells and showed higher levels of CD40 protein and luciferase signals in the presence of lipofectamine (**Fig 7E and 7F**). The increased CD40 levels stimulated by parasite DNA/RNA disappeared or were greatly reduced after treatment with nucleases (**Fig 7G and 7H**). The results are consistent with the observation of reduced CD40 and STING levels in the spleen of TRIF^-/-^ mice infected with N67 parasites (**S2 Fig**), suggesting involvement of TRIF (at least) signaling in regulating CD40 expression. We also used *Plasmodium falciparum* GPI (glycophosphatidylinositol) to stimulate RAW Lucia cells at the presence of lipofectamine. Slightly increased CD40 protein level was detected when the cells were stimulated with 1 ug GPI, but not with 0.1 ug GPI (**Fig 7I and 7J**), although a small but significant increase in ISRE-driven luciferase signal was detected in RAW Lucia cells at the GPI concentrations used (**Fig 7K**). These results are also consistent with our previous observation on TLR2^-/-^, TLR3^-/-^ and TLR4^-/-^ mice after infection with N67; parasitemia from these TLR KO mice were different from those of WT on one or more days after infection with N67 parasite [8], suggesting that these receptors play a role in host response to N67 infection.

**Fig 8:**
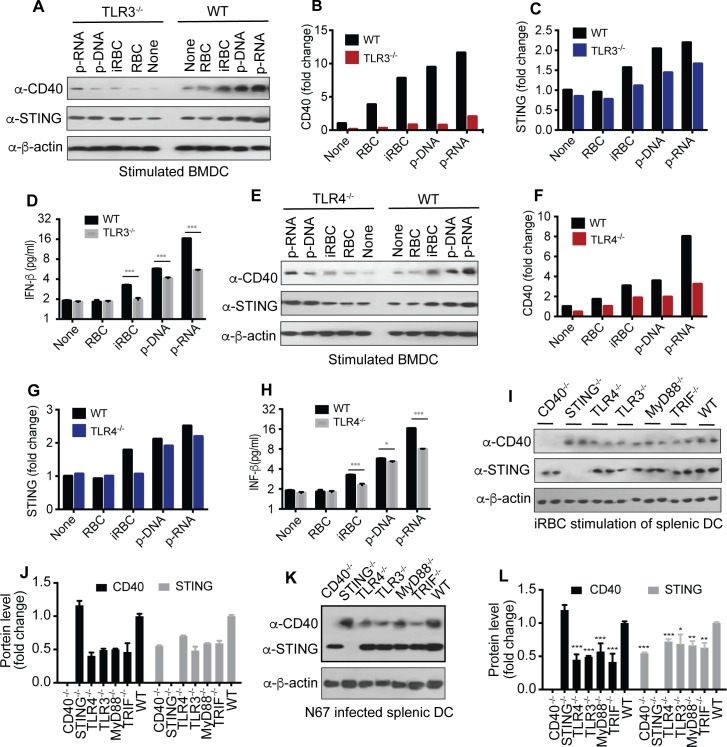
Effects of TLR signaling on CD40 and STING expressions in stimulated BMDCs and DCs from KO mice. (**A**-**D**) CD40, STING, and IFN-β expressions in BMDCs from WT and TLR3^-/-^ mice after stimulation. The cells were stimulated with the indicated agents for 24 h before harvesting proteins for Western blot. (**A**), Western blots; (**B** and **C**), quantitative signals of protein bands of CD40 and STING, respectively; (**D**) secreted IFN-β measured using ELISA. (**E-H**) the same as (**A**-**D)**, except in TLR4^-/-^ mice. (**I** and **J**) CD40 and STING expressions in purified splenic DCs from various KO mice after incubation with iRBCs for 24 h. Proteins from two mice each stimulated with iRBCs were separated on Western blot (**I**) and averaged signals were plotted in bar graphs (**J**). (**K** and **L**) CD40 and STING expressions in purified splenic DCs 4 days after infection with N67. Proteins were detected on Western blot (**K**) and scanned signals (means+s.d.) from three mice each were plotted (**L)**. *t*-test, *P<0.05; **P<0.01; ***P<0.001. For all the experiments, 5 × 10^5^ cells were used.

We next used bone marrow derived dendritic cells (BMDCs) and purified splenic DCs from CD40^-/-^, STING^-/-^, TLR3^-/-^, TLR4^-/-^, TRIF^-/-^, and MyD88^-/-^ mice to evaluate the responses to iRBC and parasite DNA/RNA. Reduction in CD40, STING and IFN-β protein levels were detected in TLR3^-/-^ (**Fig 8A–8D**) and/or TLR4^-/-^ (**Fig 8E–8H**) BMDCs after stimulation with iRBC and parasite DNA/DNA. Similarly, stimulation of purified splenic DCs from CD40^-/-^, TLR3^-/-^, TLR4^-/-^, TRIF^-/-^, and MyD88^-/-^ mice had reduced CD40 and/or STING protein levels (**Fig 8I and 8J**). Measurement of protein levels in splenic DCs of N67-infected mice day 4 *p*.*i*. (*in vivo* stimulation) showed significant reduction of CD40 and STING in DCs from TLR3^-/-^, TLR4^-/-^, TRIF^-/-^, and MyD88^-/-^ mice (**Fig 8K and 8L**). As expected, CD40 protein was increased in STING^*-/-*^ mice, whereas STING protein was decreased in CD40^-/-^. These results confirmed the observations in RAW Lucia cells and further support the involvement of the TLRs and their adaptors in CD40 and STING expression *in vitro* and *in vivo* after N67 infection.

We also performed phagocytosis of RBCs or iRBCs by bone marrow derived macrophages (BMDMs) from uninfected and day 5 infected mice *in vitro* and showed that iRBC (compared with RBC) triggered higher phagocytosis by BMDMs from both uninfected and infected mice (**S7A Fig**). Approximately 67% of BMDMs from infected mice phagocytized iRBCs (4.7% for RBC; *t*-test, *P*<0.001), whereas ~38% of BMDMs from uninfected mice contained iRBCs (14.0% for RBCs; *t*-test, *P*<0.001) 4 h after incubation (**S7A Fig**). Stimulation of BMDMs from uninfected mice with iRBCs, Poly(I:C), LPS, Pam3CSK4, and parasite DNA/RNA also induced CD40 and STING expression (**S7B Fig**). Similar results were also obtained from N67-infected mice (**S7C Fig**). Higher IFN-β levels were also detected after stimulation of BMDMs from both infected and uninfected mice with iRBC, parasite DNA/RNA and other ligands (**S7D Fig**), although some of the IFN- β measurements could be from other IFN-I signaling pathways. These results were consistent with those of RAW Lucia and DC cells described above.

## Discussion

One of the important discoveries of this study is linking the CD40 protective effect to elevated IFN-I responses in this N67 malaria parasite and C57BL/6 mouse model. CD40 is a TNF receptor superfamily member that provides critical activation signals in antigen presenting cells (APC) such as dendritic cells, macrophages, and B cells. When bound by its ligand CD40L that is transiently expressed on T cells and other non-immune cells, CD40 can activate a wide spectrum of molecular and cellular processes including the initiation and progression of cellular and humoral adaptive immunity [17, 18]. In malaria infection, CD40 was reported to be necessary for the maturation of liver DCs and for the accumulation of CD8^+^ T cells in the liver in response to the invading *P*. *yoelii* sporozoites [34]. Mice without CD40 were not able to withstand infectious challenge after immunization with a *P*. *yoelii* mutant parasite (fabb/f^-^), suggesting that CD40 signaling is a key requirement for host immunity in a lethal *P*. *yoelii*/C57BL/6 model. Additionally, CD40 expression was elevated in DCs in the immunized mice. In a non-lethal model of *Plasmodium berghei* XAT and WT C57BL/6 mice, limited protective effects were observed after administration of an agonistic anti-CD40 mAb to activate CD40 signaling, although administration of the anti-CD40 antibody to γδ T cell-deficient mice 3–10 days p.i. could help eliminate the parasites [43]. In another lethal model of *P*. *berghei* ANKA/C57BL/6 mice, host mortality was greatly decreased in CD40^-/-^ and CD40L^-/-^ mice after infection with the parasite, even though parasitemia were similar in the WT, CD40^-/-^ or CD40L^-/-^ mice [44]. In this model, mortality was attributed to the breakdown of the blood-brain barrier, macrophage sequestration, and platelet consumption. Therefore, the protective effects of CD40 KO depend on infections of specific malaria strains: Larger improvement in host survival was observed in models of early lethal infections (references 34 and 43; mice died on day 7–8 p.i.) than those of moderate or non-lethal infections (mice died ~day 15 p.i. in our N67 model and the non-lethal model in reference 44). The mechanisms of CD40-mediated protection are likely different among the models of various disease severities because dramatically different host responses were observed in infections with different parasite strains [8]. A strong day-2 IFN-I response was induced after N67 infection, leading to suppression of early parasitemia, whereas infection with N67C parasites stimulated a lethal inflammatory response with little IFN-I production [8]. In this regard, we should emphasize that the CD40/STING/IFN-I protective mechanism we observed in this N67/C57BL/6 mouse model may only apply to infections with parasite strains that can simulate strong IFN-I responses. Although the CD40/STING/IFN-I mechanism may not play an important role in some infections, it likely represents one of the possible protective mechanisms in human infections considering the potentially large number of *P*. *falciparum* strains in the field. Up-regulation of genes in IFN-I responses (ISG genes) has been associated with mild human *P*. *falciparum* malaria following an episode of severe malaria [45], suggesting that CD40/STING/IFN-I and other IFN-I protective mechanisms may play a role in infections of some human parasite strains.

DCs are the major sources of IFN-I during early malaria infection, and CD40 and CD86 were up-regulated in splenic pDCs that were dependent on TLR7 activation [8, 46, 47]. These data point to a potential link between up-regulation of CD40 expression and IFN-I production during malaria infection. Our current study showed that CD40 not only played a protective role during *P*. *yoelii* infection of C57BL/6 mice, but also regulated early IFN-I production for the first time. In our model, mice without CD40 had higher parasitemia on day 4 *p*.*i*., died earlier, and had lower levels of IFN-β than the WT mice day 2 *p*.*i*. Although there was no correlation between elevated expressions of CD40/STING and IFN-β level at day 5 *p*.*i*. *in vivo*, the down-regulation of IFN-I after day 2 *p*.*i*. was likely due to activation of negative regulators in some signaling pathways downstream of STING. Indeed, our recent study identified a large number of negative IFN-I response regulators, including several molecules (for example, *Fosl1*, *Fcgr1*, and *selenbp2*) that could down-regulate STING signaling during malaria infection [38]. However, the spike of day-2 IFN-I could affect the expression of ISGs and activate other immune pathways that could have some impact on host immune responses and survival in later phases of infection. The WT and CD40 KO mice also had similar parasitemia after day 8, but the CD40 KO mice died earlier. In the *P*. *berghei* ANKA/C57BL/6 model, better survival rates were found in CD40^-/-^ and CD40L^-/-^ mice after infection with the parasite, despite similar parasitemia in the WT, CD40^-/-^ or CD40L^-/-^ mice [44]. Similarly, mice infected with N67 and N67C parasites had almost identical parasitemia before day 5 p.i.; mice infected with N67C died on day 7, but those infected with N67 suppressed parasitemia to below 10% [8]. These observations suggested that the main cause of host death was likely due to over-responses of host immunity, not parasitemia. Our results are consistent with most of the previous studies suggesting a protective role of CD40 during malaria infections, although its functional roles in different malaria models could be different, and provide a new mechanism on CD40 mediated protection through IFN-I production during early phase of malaria infection.

The second important discovery of this study is linking the increased CD40 expression to higher STING protein level that greatly elevates the IFN-I response. A large number of studies have been done on CD40; however, the previous studies on CD40 have focused on inflammatory and adaptive immune responses to infection, not stimulation of IFN-I in early innate response. CD40 is best known for its interaction with CD40L expressed on CD4^+^ T cells and the subsequent signaling events leading to activation of NFκB and NFκB-like transcription factors and secretion of Th1 cytokines such as IL-12 and IFN-γ and to recruitment and priming of CD8^+^ T cells [17, 18]. CD40-CD40L ligation regulates production of various inflammatory cytokines such as MIP-1α, TNF-α, IL-8, and IL-12 by DCs and IL-1α, IL-1β, TNF-α, IL-6, and IL-8 by monocytes. CD40-mediated signaling leads to the transcription of host defense genes against pathogens through activation of NFκB, MAPK (Mitogen-Activated Protein Kinase) and STAT3 (Signal Transducers and Activators of Transcription-3) pathways [19, 48]. Recently, a role of CD40 in regulating IFN-β expression through canonical NFκB and non-canonical NFκB signaling pathways leading to recruitment of p52 to the IFN-β promoter was described, although the fold changes in IFN-β level mediated through the NFκB signaling were relatively small [27]. Using *in vitro* transfection of cell lines, here we showed that over expression of CD40 in 293T or RAW Lucia cells increased STING protein levels, leading to highly elevated luciferase signals driven by ISRE and IFN-β promoters. The fold changes in luciferase signals in CD40+STING co-transfected cells were much higher than those transfected with CD40 or STING alone, suggesting that CD40 can greatly enhance STING-mediated IFN-I responses to infections. The role of CD40 in enhancing STING protein level was also confirmed using purified splenic DCs or total splenic cells from WT and CD40^-/-^ mice after N67 infection. Additionally, phagocytosis of iRBCs or stimulations of BMDMs and BMDCs from infected or uninfected mice with parasite DNA/RNA also increased CD40 and STING protein levels. These *in vitro* and *in vivo* data establish a relationship of increased CD40 and STING expression. The protective roles of IFN-I in malaria infections have been reported [8–10], including injection of recombinant IFN-α [49]. Our observations of CD40 affecting STING expression and IFN-I production in the early hours of infection add a new function to CD40, in addition to its known functional roles in CD8+ T-cell activation and IgG isotype switching during host adaptive response. At day 11–15 *p*.*i*., CD40-CD40L ligation, activation of NFκB in APCs, and recruitment of CD8+ T-cells, as reported previously [34], were likely to play a role in host survival. It would be interesting to investigate how the spike of day 2 IFN-I affects the adaptive immune response, including the effects of higher CD40 level on T-cell activation and isotype switching. Proper activations of T-cells and B-cells for antibody production are critical for immune protection and host survival in later phases of infection.

Interestingly, whereas CD40 could increase STING expression and greatly enhance IFN-I production, the presence of high levels of STING appeared to decrease CD40 protein levels both in transfected cells and in mice infected with malaria parasite N67, possibly through degradation of the N-terminus of CD40. Therefore, increased STING expression may dampen CD40 mediated inflammatory responses and damage to the host, while promoting a strong IFN-I response to control parasitemia. The negative regulatory role of STING in suppressing inflammation in systemic lupus erythematosus (SLE) was reported recently [50]; our observations here add another mechanism explaining the role of STING in regulating inflammatory responses.

Many autoimmune diseases are associated with high levels of IFN-I and interferon induced genes (ISGs), particularly IFN-I produced by over-activation of STING signaling pathways [36, 37]. Interestingly, high levels of CD40 and CD40L or engagement of CD40/CD40L are also associated with the same sets of autoimmune diseases such as SLE and Aicardi-Goutieres syndrome (AGS) [28, 51]. However, the functional roles of CD40 in autoimmune and inflammatory diseases have been largely attributed to CD40-CD40L interaction that mediates T-dependent B cell responses and T cell priming [29]. Our observation of CD40 enhancement of STING mediated IFN-I production provides an alternative mechanism that may unite these two hallmarks of autoimmune diseases. Higher expression of CD40 could increase STING level in DCs and greatly elevate IFN-I production, which may influence the susceptibility to autoimmune and inflammatory diseases in addition to traditionally known pathways of T- and B-cell activation.

To investigate how the signal from CD40 was relayed to the downstream pathways leading to higher STING level, we constructed plasmids with various mutations in the cytoplasmic TRAF binding domains of CD40 and showed that the TRAF binding domains played a role in the increased STING level and IFN-I activity. Mutations in both TRAF2/3 and TRAF6 binding domains could affect luciferase activities driven by IFN-β promoter; however, when co-transfected with STING, the effect of mutations in the TRAF6 binding domain disappeared. The mutations in the TRAF binding domains also affect STING protein level, consistent with the observations showing increased CD40 and STING proteins after co-transfection of plasmids containing genes encoding both molecules and decreased STING protein in CD40^-/-^ mice after parasite infection. As expected, the mutations in the TRAF binding domains also influenced NFκB mediated signaling. In particular, introduction of TRAF3 greatly reduced CD40-mediated NFκB signaling, which was consistent with a role of TRAF3 in regulating NIK protein level. TRAF3 degradation is necessary for the cell to activate the alternative NFκB pathway [40, 41], and higher expression of TRAF3 will inhibit the alternative NFκB signaling. Overexpression of TRAF2 and TRAF3 also inhibited IFN-β production mediated by CD40 directly or by CD40-enhanced STING signaling. These observations suggest a role of TRAF2 and TRAF3 in regulating levels of STING protein and IFN-I. Both mutations in CD40 TRAF2/3 binding domains and overexpression of TRAF2/TRAF3 may lead to more ‘free’ TRAF2 and TRAF3 not associated with CD40, resulting in the observed lower levels of STING and IFN-β. On the other hand, increased CD40 levels will bind more TRAF2/TRAF3 and reduce the levels of ‘free’ TRAF2/TRAF3, resulting in higher STING and IFN-β levels because overexpression of TRAF3 led to STING degradation (**Fig 5**). It has been shown that Sendai virus (SeV) infection increased K48 and K63 ubiquitination of TRAF3/6 after recruitment of cIAP1/2 to mitochondrial associated TRAF3, which triggered IFN-I induction mediated through MAVS/VISA signaling [52]. However, viral infection or cIAP1/2 overexpression did not cause noticeable degradation of TRAF3/6, and activation of downstream kinases such as TBK1 and TAK1 were proposed. Our data suggest a mechanism of reduced availability of TRAF2 and TRAF3 after binding to elevated level of CD40 and decreased STING ubiquitination and degradation, leading to higher STING level and IFN-I production. We showed that CD40 and STING could be co-IP when cells were transfected with TRAF2, TRAF3 or TRAF6, suggesting the presence of a CD40/TRAF/STING complex. This direct contact of the molecules may explain the reduced STING ubiquitination after CD40 expression, which could be mediated by TRAF2 and/or TRAF3 directly or by recruiting other enzymes with ubiquitinase activities. Reduced ubiquitination of STING at a site such as K48 may lead to higher protein levels. Our data suggest that TRAF3 plays an important role in STING stability; however, TRAF molecules are known to play a role in many signaling pathways [53], and any change in the TRAF protein levels in the cell will likely affect more than one pathways, which will require additional investigations.

Another interesting question is how CD40 is activated by malaria ligands. Some TLRs such as TLR2 and TLR4 have MyD88-dependent and MyD88–independent pathways, depending on cellular locations of the TLRs and adaptors recruited [54, 55]. When TLR4 is located at the plasma membrane, activation of the MyD88-dependent pathway leads to production of proinflammatory cytokines; whereas the MyD88-independent signaling pathway is involved when TLR4 is at the endosome [54]. The MyD88-independent pathway signals through a TRIF-related adaptor molecule (TRAM), TRIF and IRF3, which leads to IFN-I production [55–57] and/or activation of CD40, CD54 (ICAM1) and CD86 [58]. Additionally, LPS has been shown to stimulate CD40 expression through TLR4 signaling that involved activation of both NFκB and signal transducer and activator of transcription1α (STAT-1α) in macrophages and microglia [42]. Various TLRs are known to play a role in host response to malaria infections [59, 60]. Consistent with these reports, we showed that over expressions of TLR1/2, TLR3, and TLR4 followed by stimulations with their ligands [Pam3CSK4, poly(I:C), and LPS, respectively] could increase CD40 protein levels as well as luciferase signals driven by ISRE promoter in 293T cells. Consistently, stimulations of BMDMs and BMDCs with TLR ligand poly(I:C) and LPS, parasite DNA/RNA, and iRBCs increased CD40/STING levels (**S7 Fig**), and compared with cells from WT, stimulations of BMDCs from TLR3^-/-^ and TLR4^-/-^ mice with parasite DNA/RNA and iRBCs had reduced CD40/STING proteins and IFN-β (**Fig 8).** Additionally, splenic DCs from TLR3^-/-^, TLR4^-/-^, TRIF^-/-^, and MyD88^-/-^ had reduced CD40 and/or STING protein levels after *in vitro* stimulations with iRBC or *in vivo* N67 infection. These data support the involvement of TLR3 and TLR4 in CD40/STING expressions, although we still do not understand how TLR4 KO affects CD40/STING/IFN-β expression. TLR2 and to a lesser extent TLR4 were shown to mediate *P*. *falciparum* GPI recognition and signaling leading to proinflammatory responses [61]. Patients with severe and mild malaria also showed increased surface expression of TLR2 and TLR4 on CD14^+^ monocytes and cDCs and decreased intracellular expression of TLR9 on pDCs [59, 62]. However, these reported TLR signaling pathways may or may not lead to increased expression of CD40, STING, and IFN-I. TLR9 recognizes unmethylated CpG dinucleotides and signals mostly through the MyD88/NFκB pathway. TLR9 was reported to play an important role in the regulation and development of protective immunity to malaria [63, 64]. Although our data showed that stimulation of TLR9 only slightly increased CD40 expression in 293T cells *in vitro*, and we did not have TLR9^-/-^ mice to confirm the role of CD40 expression *in vivo*, the effects of MyD88 KO on CD40/STING expression in the infected splenic DCs suggested a minor role of TLR9 in parasite DNA recognition and CD40 expression. Parasite DNA can be converted into 5’ppp-RNA and recognized by cytosolic RNA sensors [8]. An unknown DNA sensor was reported to recognize AT-rich parasite DNA [7]. This receptor could be the recently identified cGAS [65] or an additional unknown receptor that may stimulate CD40 expression. Additional investigations are necessary to identify parasite DNA receptor(s) that can stimulate CD40/STING expression during malaria infection.

Importantly, we also showed that iRBCs and parasite-derived materials including nucleic acids and possibly GPI could also increase CD40 levels and/or luciferase signals driven by ISRE promoter, although potential DNA contamination in GPI preparations might contribute to the observed activity [46, 64]. Furthermore, there were good correlations between CD40/STING protein levels and ISRE driven luciferase signals after poly(I:C), LPS, and iRBC stimulations, indicating a causative effect of CD40/STING protein levels and IFN-I/ISG responses. These data suggest that these parasite molecules can first activate CD40 expression through TLR/TRIF and other signaling pathways, leading to increased STING and IFN-I expression. Although the observations of changes in CD40 expression after LPS and iRBC stimulations or TLR4 KO suggested potential involvement of TLR4 (maybe through TLR4/TRAM/TRIF/IRF3 signaling) in CD40 expression, we are not sure of the specific parasite ligands for TLR4. Parasite GPI could be a potential TLR2/4 ligand [61]. Interestingly, we also showed that *P*. *falciparum* GPI could stimulate low level, but significant, ISRE derived luciferase activity in RAW Lucia cell, although we could detect a small difference in CD40 protein level only at the 1 μM GPI group (**Fig 7J**). The change in luciferase signal at the 0.1 μM GPI group was very small, and Western blot might not be sensitive enough to detect the small difference in protein band intensity. The luciferase signals could also be produced by pathways other than CD40/STING signaling. Our data also suggest that parasite nucleic acids can act as TLR3 ligands because TLR3 KO significantly affects CD40 and STING protein levels after parasite DNA/RNA stimulations.

Based on the observations in this study, we can construct some preliminary signaling pathways for the elevated IFN-I response mediated by the interaction of CD40 and STING (**S8 Fig**) during early malaria infection, at least for the N67/C57BL/6 model. First, phagocytosis of iRBCs releases parasite materials such as nucleic acids and possibly lipids/GPI that are recognized by TLRs (2, 3 and 4), which triggers signaling mostly through TRIF dependent pathways leading to increased CD40 protein level. The increased CD40 expression leads to reduced STING ubiquitination and enhanced-levels of STING through binding additional TRAFs, particularly TRAF3. Increased CD40 expression binds more TRAF2 and TRAF3 and diminishes the pools of ‘free’ TRAF2 and TRAF3, leading to reduction of STING ubiquitination and degradation. Increased STING level can then greatly facilitate IFN-I production leading to suppression of early parasitemia and better host survival. In addition to demonstrating the protective role of CD40 in malaria infection by increased IFN-I production, this study reveals an unknown function of CD40 and TRAF2/TRAF3 in regulating STING and IFN-I expression and connects several innate signaling pathways involving TLRs, CD40, STING and TRAFs. Of course, this CD40/STING/IFN-I signaling pathway is just one of many protective mechanisms the host mounts against the parasites, and a complete resolution of malaria infection will require a well-balanced response involving a large number of molecules. Our observations may also provide important information for better understanding of the molecular mechanisms underlying many autoimmune diseases such as SLE and AGS that have been associated with STING-mediated high IFN-I levels and/or CD40-CD40L meditated T- and B-cell activation and inflammation. Our data suggest an alternative mechanism that CD40 may contribute to autoimmune diseases through elevated activities of STING and IFN-I production.

## Materials and Methods

### Ethics statement

All animal procedures were performed in accordance with the approved protocol (approval #LMVR11E) by Institutional Animal Care and Use Committees (IACUC) at the National Institute of Allergy and Infectious Diseases (NIAID) DIR ACUC following the guidelines of the Public Health Service Policy on Humane Care and Use of Laboratory Animals and AAALAC.

### Parasites and mice

The *P*. *y*. *nigeriensis* used in this study was described previously [66]. Parasitemia were counted from Giemsa stained thin blood smears using a light microscope.

C57BL/6 mice, 5- to 8-weeks-old, from Charles River Laboratories were injected with 1×10^6^ iRBCs as described [66, 67]. The genetic KO mice (CD40^-/-^, TLR3^-/-^, TLR4^-/-^, TRIF^-/-^, MyD88^-/-^, STING^gt/gt^) were from the Jackson Laboratory.

### Antibodies and related reagents

Anti-CD40 N-terminus (H-120; sc-9096) and anti-CD40 C-terminus (C-20; sc-975) were purchased from Santa Cruz Biotechnology (Dallas, TX); Anti-β-actin (A2228) was from Sigma (St. Louis, MO); anti-DDK mouse mAb (8146), anti-HA rabbit mAb (3724), and anti-STING (3647) were from Cell Signaling Technology (Danvers, MA). Primary antibody binding was visualized using horseradish peroxidase (HRP)-conjugated secondary antibodies specific for mouse or rabbit IgG (Sigma). The anti-Myc (mouse mAb) and anti-HA (rabbit mAb) were used in immunofluorescence microscopy (IFA). Secondary antibodies used for IFA were Alexa Fluor 488 goat anti-mouse IgG and Alexa Fluor 594 goat anti-rabbit IgG (Abcam, Cambridge, MA). DMXAA, 2’3’-cGAMP, Pam3CSK4, CpG (ODN 2395) were from InvivoGen. Poly(I:C), LPS, Poly(dAdT) were from Sigma. *P*. *falciparum* GPI was prepared as described [68].

### Cells and cell culture

Human embryonic kidney 293T (ATCC, Manassas, VA), Hela (ATCC, Manassas, VA), RAW Lucia ISG cells (InvivoGen, Carlsbad, CA) and DC2.4 (ATCC, Manassas, VA) were maintained in DMEM containing 10% heat-inactivated FBS, 50 units/ml penicillin and 50 mg/ml streptomycin. BMDMs were collected from mouse femurs and tibiae. Cells were cultured in DMEM containing 10% FBS, antibiotics, and 30% L929 supernatant. The cells were grown for 7 days with addition of 10 ml fresh DMEM/L929 on day 4. Mature cells were harvested using Nunc Cell Scrapers and plated into 24-well plates for further experiments. BMDCs were obtained by flushing bone-marrow cells from the marrow cavities of femurs and tibiae of C57BL/6 WT or different KO mice. Erythrocytes were depleted with ACK lysis buffer (Thermo Fisher Scientific), and the remaining cells were cultured (as day 0) at 1 × 10^7^ cells/petri dish in the presence of murine recombinant GM-CSF at 20 ng/mL (PeproTech, Rocky Hill, NJ) in complete medium (DMEM with 10% heat-inactivated FBS and penicillin/streptomycin antibiotics). The cultures were replenished with fresh medium on day 3. At day 6, primary/immature BMDCs were harvested and 2 x 10^5^ cells per well (of 24-well plate) were seeded for subsequent ligand or iRBCs stimulation. DCs from spleen were purified using a EasySep Mouse Pan-DC Enrichment Kit (STEMCELL Technologies, Cambridge, MA). Mouse IFN-β was detected using an ELISA kit according to the manufacturer's protocols (PBL Biomedical Laboratories, Piscataway, NJ).

### Immunofluorescence assay (IFA)

Cells cultured in glass bottom dishes were fixed with 4% formaldehyde in PBS, pH = 7.3 for 15 min, followed by permeabilization with 0.5% Triton X-100 for 15 min at room temperature (RT). Primary anti-DDK mouse mAb for CD40-DDK staining and anti-HA rabbit mAb for STING-HA staining were added (1:2000 dilution) for 1 h at RT after blocking with washing buffer (3% BSA in PBS) for 1 hour. The samples were further stained with Alexa Fluor 488 goat anti-mouse IgG or Alexa-594 goat anti-rabbit IgG, respectively, followed by 3X washes, and were mounted using ProLong Gold anti-fade reagent with DAPI (Life technologies, Carlsbad, CA). Images were captured using Zeiss LSM 780 confocal microscope.

### Cell transfection and luciferase reporter assay

293T cells (2 × 10^5^) were plated in 24-well plates and transfected using lipofectamine 2000 (Thermo Fisher Scientific). Twenty ng of renilla luciferase reporter plasmid and 50 ng of firefly luciferase reporter plasmids were transfected together with indicated expression plasmids. Luciferase activity was measured 24 h (or as indicated) after transfection using the Dual-Glo Luciferase Assay System (Promega, Madison, WI) with a BMG FLUOstar OPTIMA microplate reader (BMG LABTECH, Cary, NC). An Amaxa nucleofector kit V was also used for transfection according to the manufacturer's protocols (Lonza, Walkersville, MD)

### Co-immunoprecipitation and immunoblot analysis

The STING-HA expression plasmids were kindly provided by Dr. Russell E. Vance1 (University of California, Berkeley, California) and have been previously described [69]. CD40-Myc was cloned into pCMV6-entry vector as described below. For immunoprecipitation, whole-cell extracts were prepared after transfection or stimulation with appropriate ligands, followed by incubation with anti-HA or anti-Myc magnetic beads (Thermo Fisher Scientific, Rockville, MD). Co-IP was performed according to the manufacturer’s instructions and subsequently analyzed on Western blot. Cell lysates or immunoprecipitated complexes were separated on SDS-PAGE gels and transferred to PVDF membranes (Thermo Fisher Scientific). Membranes were incubated with primary antibodies followed by HRP-conjugated secondary antibodies. Bound HRP-labeled antibodies were visualized using SuperSignal West Dura Extended Duration Substrate (Thermo Fisher Scientific).

### Construction of plasmids for mutagenesis of TRAF binding domains

Mouse *cd40* sequence was PCR-amplified using a MGC Mouse cDNA library (CloneId:4018221) as template. PCR primers were synthesized based on GenBank accession number BC029254.1. The *cd40* cDNA was cloned into the *Sgf*I and *Mlu*I sites of pCMV6-entry vector (Origene, Rockville, MD) after PCR amplification using primers: 5’atctgccgccgcgatcgcCATGGTGTCTTTGCCTCGGC 3’ and 5’ tcgagcggccgcgtacgcgtGACCAGGGGCCTCAAGGC 3’. A C-terminal DDK/Myc tagged *cd40* was expressed under the control of CMV promoter. Point mutations in the cytoplasmic tail of CD40 were generated from this CD40-DDK/Myc expression vector using QuikChange II Site-Directed Mutagenesis Kit (Agilent Technologies, Santa Clara, CA) according to the manufacturer’s instructions. The following primers were utilized for the site-directed mutagenesis reactions with the desired mutations underlined: 5' CCAGTGCAGGAGGCGCTGCACGGGT 3' and its complementary oligonucleotide 5' ACCCGTGCAGCGCCTCCTGCACTGG 3' to generate T255A for mT2/3; 5' CGGGTGTCAGCCTGTCACAGCGGAGGATGGT 3' and its complementary oligonucleotide 5' ACCATCCTCCGCTGTGACAGGCTGACACCCG 3' to generate Q264A for mbx2; 5' CTCGACGGCAAGATCCCGCGGCGATGGAAGATTATCCCG 3' and its complementary oligonucleotide 5'CGGGATAATCTTCCATCGCCGCGGGATCTTGCCGTCGAG 3' to generate Q238A and E239A for mT6;

### Collection of *P*. *yoelii* schizonts for phagocytosis

Blood samples of 1.5–2 ml were collected from two N67- infected mice (20–40% total parasitemia) and synchronized as described [70]. Briefly, collected blood was immediately added to 5 ml anticoagulant solution (1.5% sodium citrate, 0.9% NaCl). The blood sample was passed through a NWF filters (Zhixing Bio, Bengbu, China) to remove white blood cells [71]. The flow-through containing RBCs were centrifuged at 2000 rpm for 5min, and the pellet was suspended in RPMI 1640 culture medium [RPMI 1640 1000 ml, NaHCO_3_ (7.5%) 30 ml, gentamycin (10 mg/ml) 1ml, 20% (vol/vol) FBS] and distributed into culture flasks. The flasks were incubated at 37°C for 16 h in a humidified CO2/air incubator with shaking (60 rpm). The cultures were centrifuged at 2,500 rpm for 5 min, and the pelleted RBCs were re-suspended in 2 ml of PBS with 5% FBS. Two ml culture suspension was carefully placed on top of 5 ml 72% Percoll/NaCl solution (6.5ml Percoll, 0.72 ml of 1.5 M NaCl, 2.8 ml of 0.15 M NaCl) and centrifuged at 450 *g* in a swing-out rotor at RT for 20 min. Schizonts at the interface were transferred to a 50-ml tube. Control RBCs were incubated and treated in the same way without schizont purification.

### Phagocytosis assay

Approximately 2×10^6^ RAW Lucia cells were plated into wells and grown to 70–80% confluence before addition of 2×10^7^ iRBCs or RBCs, respectively. The cells were incubated at 37°C and 5% CO_2_, and samples were collected for the flow cytometry after 1 h or 8 h incubation. Flow cytometry analysis was performed on a BD FACSCalibur equipped with a 488 nm laser and three detection channels (FL-1: 515–550 nm, FL-2: 575–620 nm, FL-3: >630 nm). Data acquisition and analysis was done using FlowJo. RAW Lucia cells were gated and plotted based on the forward scatter (FSC) versus side scatter (SSC). For staining of RBCs, 5×10^7^ RBCs in 1 ml PBS were incubated with 5 μM CFDA-SE (Thermo Fisher Scientific) at RT for 5 min before washes and flow cytometry counting. Counts from phagocytosis of uninfected RBCs were excluded by gating the same areas from RBC controls.

### Ubiquitination assay

For analysis of STING ubiquitination, 293T cells were transfected with plasmids expressing STING with C-terminal Myc tag and CD40 or empty vector as well as HA-tagged WT ubiquitin or ubiquitin mutants containing only one lysine at specific position (K6, K11, K27, K29, K33, K48, K63). The mutant ubiquitin plasmids were obtained from Addgene originally deposited by Sandra Weller as described [72]. Twenty hrs after transfection, cell lysates were immunoprecipitated using anti-Myc beads, followed by immunoblot analysis with anti-HA to detect ubiquitinated STING.

## Supporting Information

S1 Fig Increased mRNA expression of CD40 and interferon induced genes after *Plasmodium yoelii nigeriensis* infection. (**A**) Clusters of up-regulated expression of genes (compared with those of uninfected) in GO-terms related to type I interferon response in mouse spleens after infection with *P*. *y*. *nigeriensis* N67 and N67C. The original genome-wide gene expression data were obtained using a microarray and were reported in [38]. (**B**) Log_10_ ratios of selected gene expression in mouse spleens infected with *P*. *y*. *nigeriensis* N67 or N67C parasites. Higher expression levels were detected in mice infected with N67 than those infected with N67C although mice infected with both parasites had greatly increased expression of the selected genes.
(PDF)

S2 Fig Effect of gene knockout on CD40 expression during malaria infection. (A) CD40 and STING protein levels in infected mouse spleen cells detected on Western blot. Mice with specific gene knockout as indicated were infected with *Plasmodium y*. *nigeriensis* N67, and proteins from spleen cells 4 days post infection (or day 5) were harvested and detected on immunoblots using anti-CD40 or anti-STING antibodies. (B) Relative protein levels after justification for protein loading variation. Protein bands were scanned and quantitated, and signal ratio of each protein band was obtained after dividing the signal intensity of a specific protein band by that of its corresponding β-actin. The proteins were from a single mouse; similar results were obtained from DC cells of additional mice (**Fig 8**). WT, wild type mice; name_U., WT or KO mice, uninfected.
(PDF)

S3 Fig Generation of CD40 mutants with amino acid substitutions at the TRAF binding domains. (**A**) Partial CD40 amino acid sequence with amino acid substitution sites (in red) in the TRAF binding domains and Box 2. (**B**) Aligned nucleotide sequences showing expected nucleotide substitutions (in red) after DNA sequencing. (**C-E**) Electropherograms showing the expected nucleotides (arrows) in each TRAF binding domain from Sanger dye terminator sequencing.
(PDF)

S4 Fig Effects of TRAF molecules on CD40 and/or STING mediated NFκB or IFN-β signaling. (**A-C**) Luciferase signals driven by NFκB promoter with (blue) or without (black) TRAF molecules. Although TRAF2 and TRAF6 had some effects on NFκB activation, TRAF3 was the one that has a clear negative effect on CD40 mediated activities. (**D-F**) Luciferase signals driven by IFN- β promoter with (red) or without (black) TRAF molecules. TRAF2 and TRAF3 could inhibit CD40-enhanced STING activities on IFN-I production, whereas TRAF6 had opposite effect by increasing IFN- β level. For the experiments, 293T cells (2 × 10^5^) were transfected with indicated plasmids, and luciferase activities were measured 24 h after transfection. All data are means+s.d. from three experiments; *t*-test, *P<0.05; **P<0.01; ***P<0.001.
(PDF)

S5 Fig Localization of CD40 and STING proteins. (**A**) Indirect immunofluorescent assays (IFA) using the anti-HA (STING) and anti-DDK (CD40) antibodies showing no co-localization of CD40 and STING in 239T cells 24 h after transfection of plasmids encoding CD40-DDK and STING-HA. **(B**) The same experiments as in (**A**) using Hela cells.
(PDF)

S6 Fig Protein expression of CD40 and STING and ISRE driven luciferase signals after stimulations with various ligands or infected red blood cells (iRBCs). (**A**) Plots of protein band intensities of CD40 and STING and IRSE-luciferase signals 1, 8, and 24 h after Pam3CSK4 stimulation of RAW Lucia cells (2 × 10^5^). All data are means from three experiments. (**B**) The same plots as in (**A**), but stimulated with DXMAA. (**C**) Detection of CD40 protein after stimulations with various agents (as indicated). RAW Lucia cells (2 × 10^5^) were incubated with the agents for 24 h before harvesting for protein analysis. Anti-β-actin was used as protein loading control. Dosages: RAW Lucia cells: RBC (1:10); RAW Lucia cells:iRBC (1:10); DMXAA, 1 μg/ml; cGAMP, 1 μg/ml; poly(I:C), 1 μg/ml; LPS, 0.5 μg/ml; Pam3CSK4, 1 μg/ml; poly(dAdT), 1 μg/ml; CD40L, 0.5 μg/ml; IFN-Υ, 0.1 μg/ml; CD40/IFN-γ, 0.5 μg/ml/1 μg/ml. (**D**) Luciferase signals driven by ISRE promoter after 24 h stimulations of RAW Lucia cells (2 × 10^5^) with the indicated agents for 24 h. All data are means+s.d. from three experiments. The same dosages as in (**C**). (**E**) CD40 protein levels in 293T cells (2 × 10^5^) after CD40L stimulation at the indicated time and dosages.
(PDF)

S7 Fig Phagocytosis of red blood cells (RBC) and infected RBCs (iRBCs) by bone marrow derived macrophages (BMDMs) from uninfected and infected mice. (**A**) Flow cytometry counts of cells with phagocytized RBCs. BMDM prepared, as described in the Methods, were incubated with RBCs or iRBCs for 1h and 4h, and the cells were counted using flow cytometry. The figures were from a representative experiment, and the numbers within each window were average percentage of cells with phagocytized RBCs counts from three replicates. N67 iRBC is *Plasmodium y*. *nigerinesis* N67 infected RBCs. SSC, side-scattered light; CF-SE, carboxyfluorescein succinimidyl ester labeled RBCs or iRBCs (**B** and **C**), Western blot detection of CD40 and STING expression 24 h after stimulations of BMDMs from uninfected (**B**) or day-5 N67-infected mice (**C**). (**D**) Interferon β (IFN-β) in supernants after stimulations with the indicated ligands for 24 h, measured using ELISA. Note that high levels of IFN-β from the cGAMP and poly(dAdT) stimulated cells could be from direct stimulation/activation of STING or pathways of other cytosolic nucleic acid sensors. iBMDM, cells from N67-infected mice; BMDM, cells from uninfected mice. *t*-test; 1, *p*<0.05; 2, *p*<0.01; 3, *p*<0.001, comparing with those of non-stimulated (None). **, *p*<0.01 comparing with those of RBC stimulated groups. Infected and uninfected groups were compared separately.
(PDF)

S8 Fig Schematic of molecular signaling pathways leading to high level of type I interferon and better host survival. First, parasite nucleic acids (or GPI/lipids) activate TLRs after phagocytosis of infected red blood cells (iRBCs), leading to activation of TRIF dependent signaling pathways. Second, TLR signaling stimulates and increases CD40 expression. Third, increased CD40 expression leads to higher STING protein level, possibly through changes on STING ubiquitination and degradation that is influenced by levels of TRAF2/3. Fourth, higher STING level greatly increases IFN-I production during early infection. Finally, higher levels of IFN-I suppresses day 4 parasitemia and enhances host survival. This CD40/STING mediated enhanced IFN-I response may also play a role in some autoimmune disorders. Note that the high level of IFN-I triggers many negative regulators that quickly suppress the production of IFN-I.
(PDF)
