# Supplementary figures and images for: Increased CD40 Expression Enhances Early STING-Mediated Type I Interferon Response and Host Survival in a Rodent Malaria Model
Source: PLoS Pathog. 2016 Oct 7;12(10):e1005930. doi: 10.1371/journal.ppat.1005930 (PMC5055354; doi:10.1371/journal.ppat.1005930)

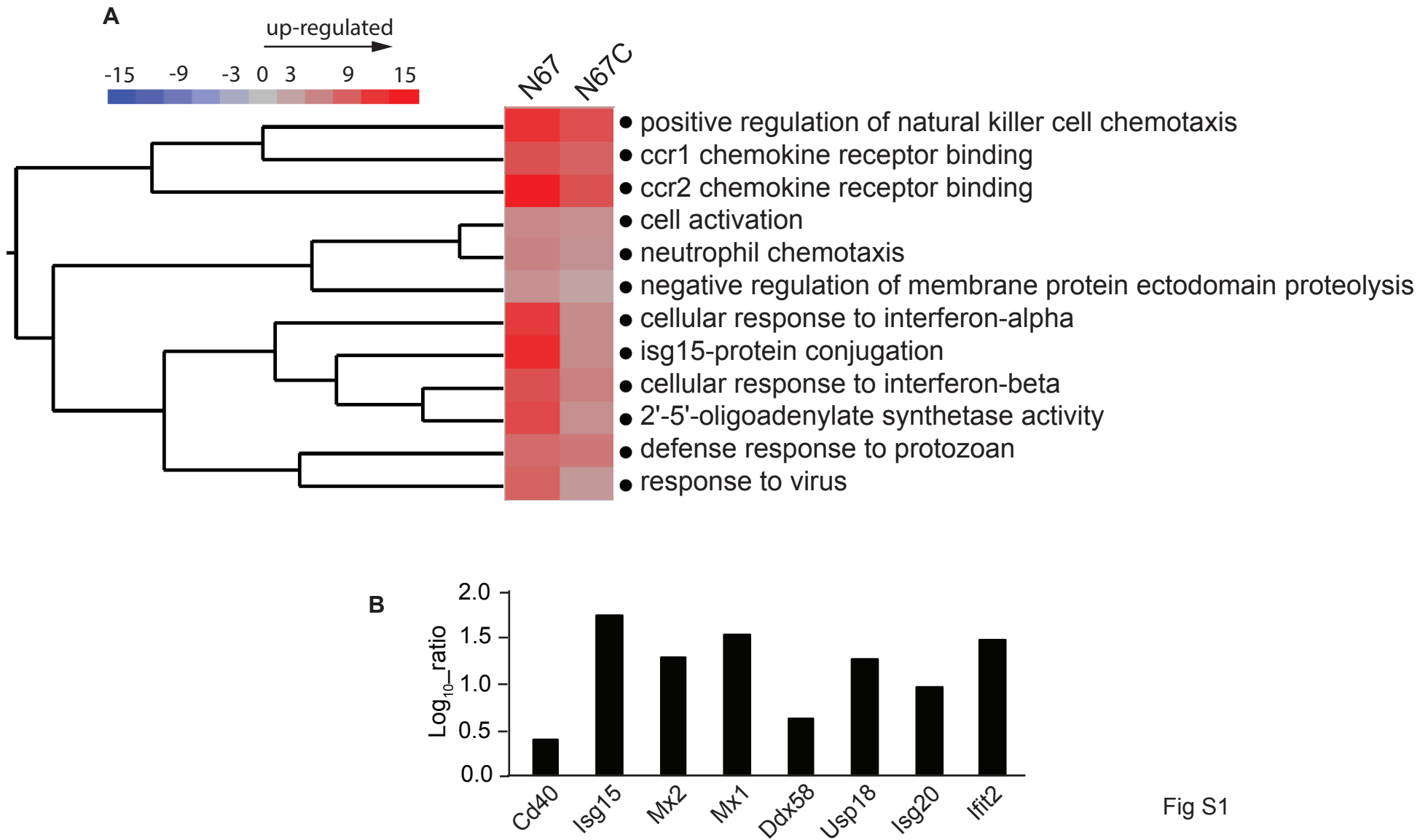

Fig S1

Supplement: S1 Fig — (A) Clusters of up-regulated expression of genes (compared with those of uninfected) in GO-terms related to type I interferon response in mouse spleens after infection with P. y. nigeriensis N67 and N67C. The original genome-wide gene expression data were obtained using a microarray and were reported in [38]. (B) Log10 ratios of selected gene expression in mouse spleens infected with P. y. nigeriensis N67 or N67C parasites. Higher expression levels were detected in mice infected with N67 than those infected with N67C although mice infected with both parasites had greatly increased expression of the selected genes. (PDF) [file ppat.1005930.s001.pdf]

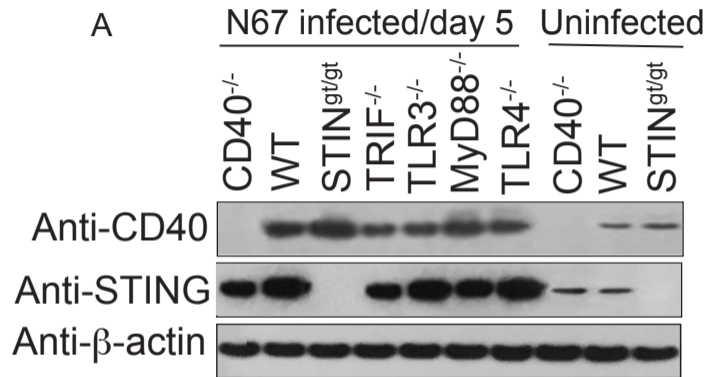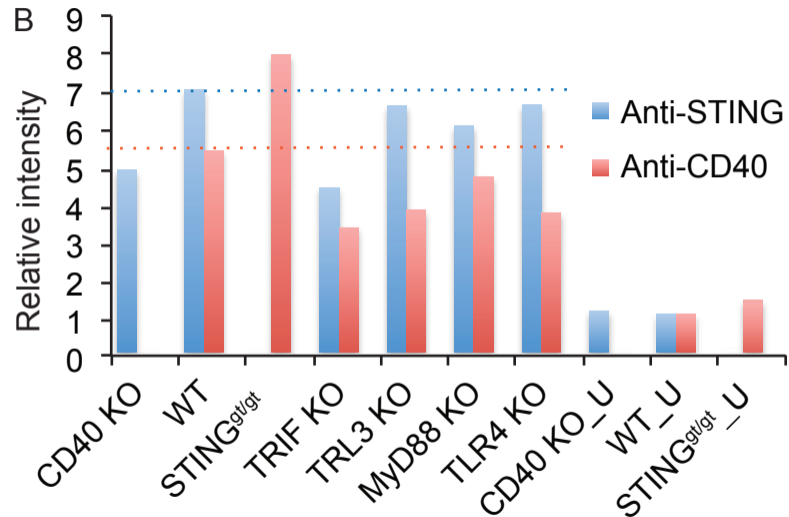

Fig S2

Supplement: S2 Fig — (A) CD40 and STING protein levels in infected mouse spleen cells detected on Western blot. Mice with specific gene knockout as indicated were infected with Plasmodium y. nigeriensis N67, and proteins from spleen cells 4 days post infection (or day 5) were harvested and detected on immunoblots using anti-CD40 or anti-STING antibodies. (B) Relative protein levels after justification for protein loading variation. Protein bands were scanned and quantitated, and signal ratio of each protein band was obtained after dividing the signal intensity of a specific protein band by that of its corresponding β-actin. The proteins were from a single mouse; similar results were obtained from DC cells of additional mice (Fig 8). WT, wild type mice; name_U., WT or KO mice, uninfected. (PDF) [file ppat.1005930.s002.pdf]

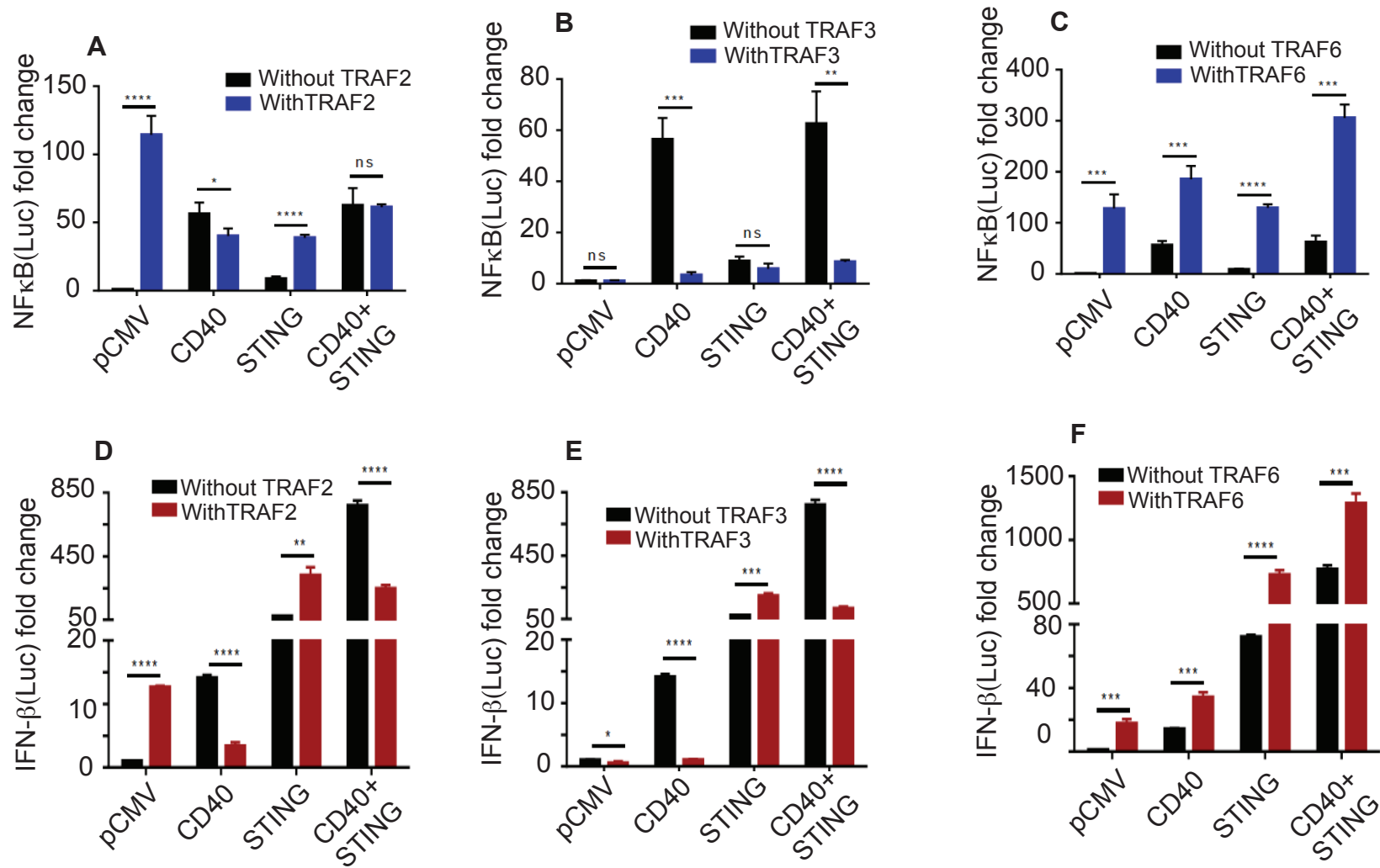

Fig S4

Supplement: S4 Fig — (A-C) Luciferase signals driven by NFκB promoter with (blue) or without (black) TRAF molecules. Although TRAF2 and TRAF6 had some effects on NFκB activation, TRAF3 was the one that has a clear negative effect on CD40 mediated activities. (D-F) Luciferase signals driven by IFN- β promoter with (red) or without (black) TRAF molecules. TRAF2 and TRAF3 could inhibit CD40-enhanced STING activities on IFN-I production, whereas TRAF6 had opposite effect by increasing IFN- β level. For the experiments, 293T cells (2 × 10^5^) were transfected with indicated plasmids, and luciferase activities were measured 24 h after transfection. All data are means+s.d. from three experiments; t-test, *P<0.05; **P<0.01; ***P<0.001. (PDF) [file ppat.1005930.s004.pdf]

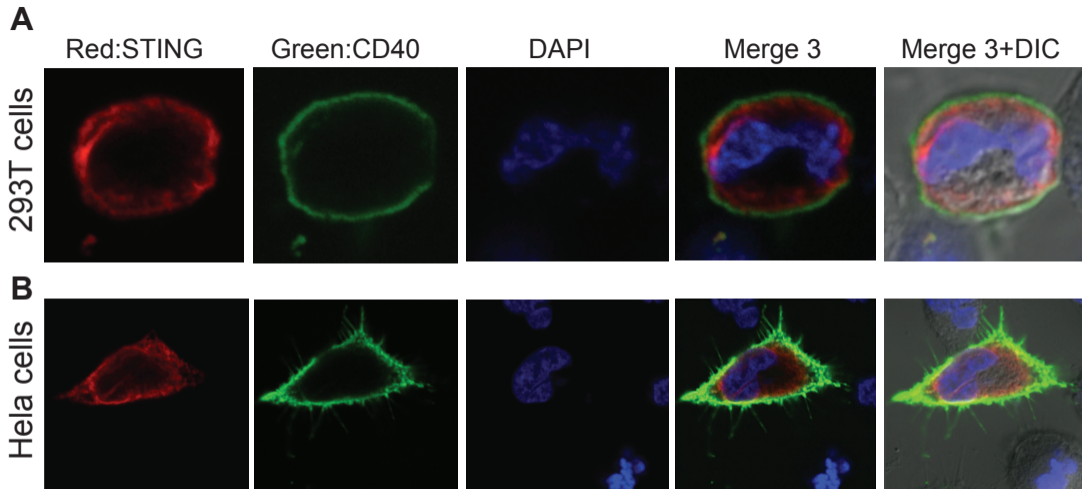

Fig S5

Supplement: S5 Fig — (A) Indirect immunofluorescent assays (IFA) using the anti-HA (STING) and anti-DDK (CD40) antibodies showing no co-localization of CD40 and STING in 239T cells 24 h after transfection of plasmids encoding CD40-DDK and STING-HA. (B) The same experiments as in (A) using Hela cells. (PDF) [file ppat.1005930.s005.pdf]

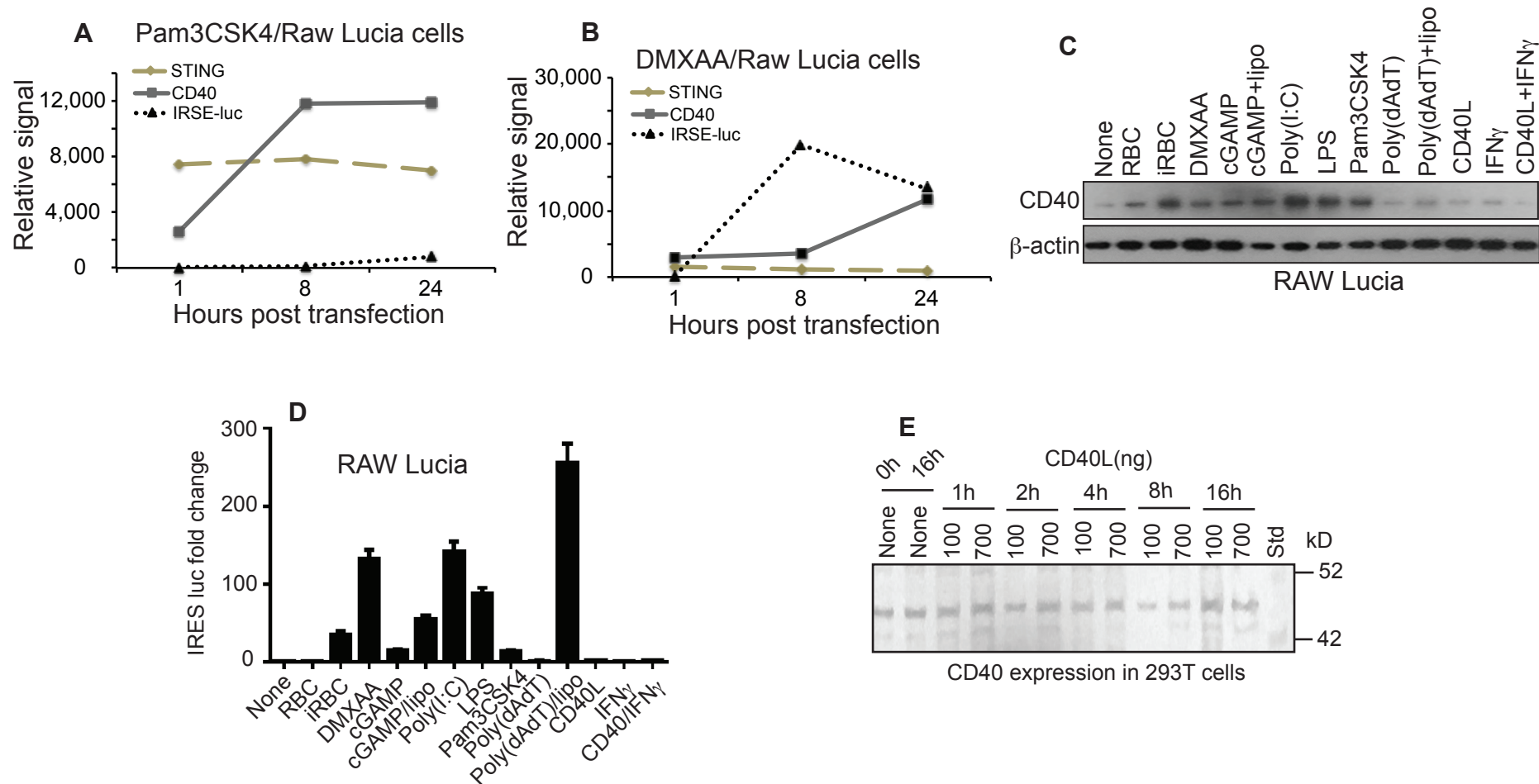

Fig S6

Supplement: S6 Fig — (A) Plots of protein band intensities of CD40 and STING and IRSE-luciferase signals 1, 8, and 24 h after Pam3CSK4 stimulation of RAW Lucia cells (2 × 10^5^). All data are means from three experiments. (B) The same plots as in (A), but stimulated with DXMAA. (C) Detection of CD40 protein after stimulations with various agents (as indicated). RAW Lucia cells (2 × 10^5^) were incubated with the agents for 24 h before harvesting for protein analysis. Anti-β-actin was used as protein loading control. Dosages: RAW Lucia cells: RBC (1:10); RAW Lucia cells:iRBC (1:10); DMXAA, 1 μg/ml; cGAMP, 1 μg/ml; poly(I:C), 1 μg/ml; LPS, 0.5 μg/ml; Pam3CSK4, 1 μg/ml; poly(dAdT), 1 μg/ml; CD40L, 0.5 μg/ml; IFN-Υ, 0.1 μg/ml; CD40/IFN-γ, 0.5 μg/ml/1 μg/ml. (D) Luciferase signals driven by ISRE promoter after 24 h stimulations of RAW Lucia cells (2 × 10^5^) with the indicated agents for 24 h. All data are means+s.d. from three experiments. The same dosages as in (C). (E) CD40 protein levels in 293T cells (2 × 10^5^) after CD40L stimulation at the indicated time and dosages. (PDF) [file ppat.1005930.s006.pdf]

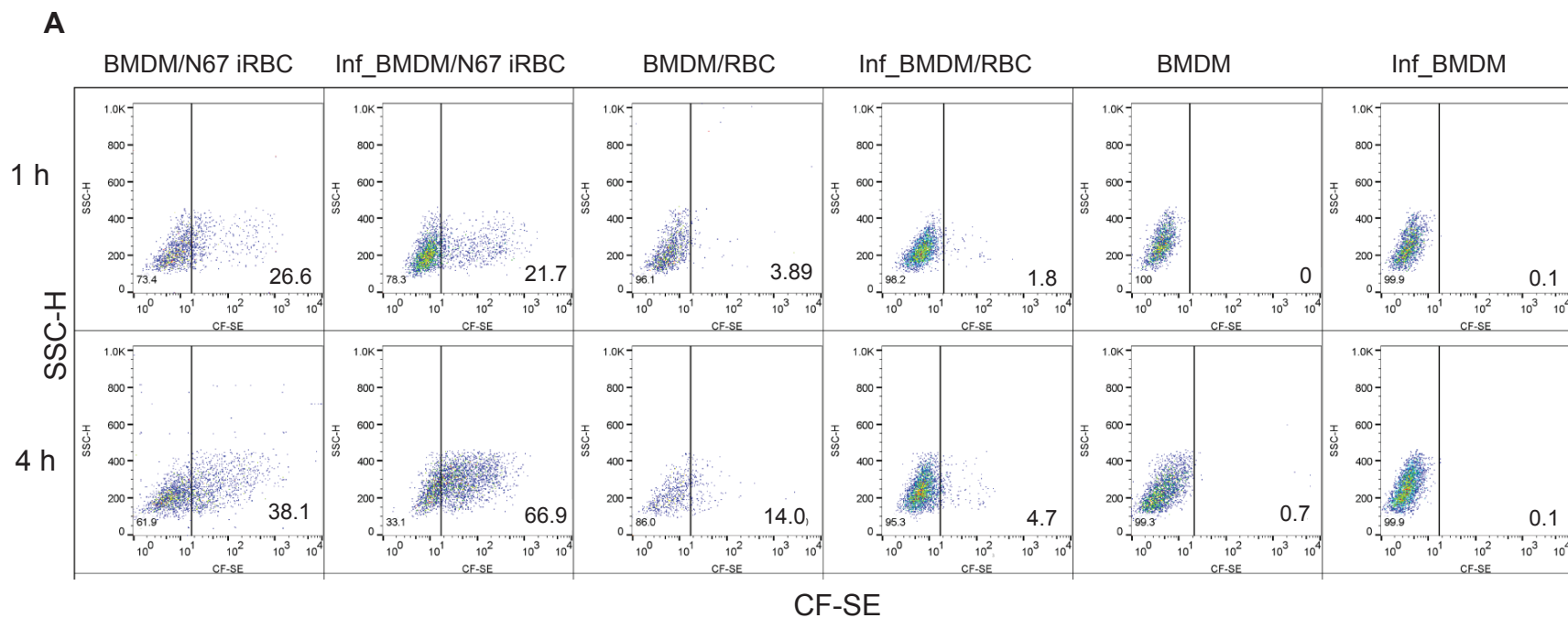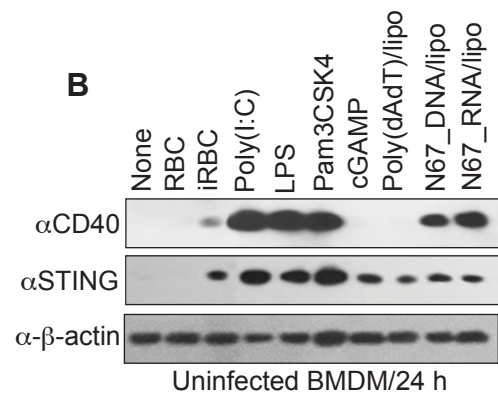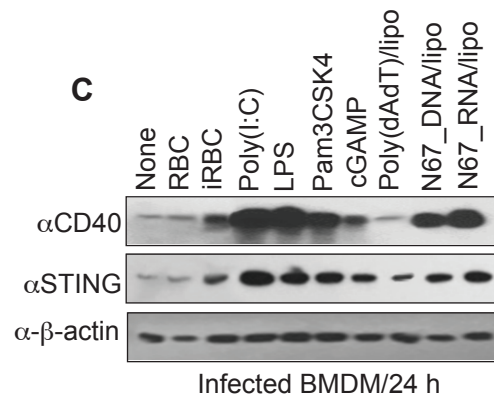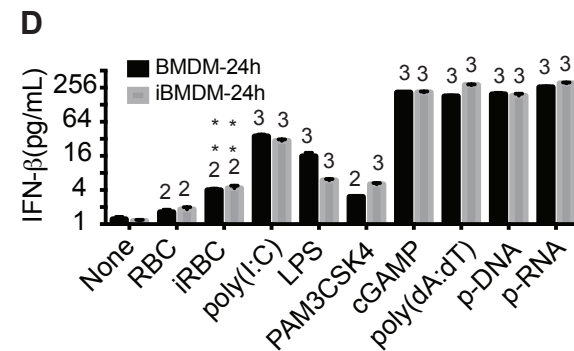

Fig S7

Supplement: S7 Fig — (A) Flow cytometry counts of cells with phagocytized RBCs. BMDM prepared, as described in the Methods, were incubated with RBCs or iRBCs for 1h and 4h, and the cells were counted using flow cytometry. The figures were from a representative experiment, and the numbers within each window were average percentage of cells with phagocytized RBCs counts from three replicates. N67 iRBC is Plasmodium y. nigerinesis N67 infected RBCs. SSC, side-scattered light; CF-SE, carboxyfluorescein succinimidyl ester labeled RBCs or iRBCs (B and C), Western blot detection of CD40 and STING expression 24 h after stimulations of BMDMs from uninfected (B) or day-5 N67-infected mice (C). (D) Interferon β (IFN-β) in supernants after stimulations with the indicated ligands for 24 h, measured using ELISA. Note that high levels of IFN-β from the cGAMP and poly(dAdT) stimulated cells could be from direct stimulation/activation of STING or pathways of other cytosolic nucleic acid sensors. iBMDM, cells from N67-infected mice; BMDM, cells from uninfected mice. t-test; 1, p<0.05; 2, p<0.01; 3, p<0.001, comparing with those of non-stimulated (None). **, p<0.01 comparing with those of RBC stimulated groups. Infected and uninfected groups were compared separately. (PDF) [file ppat.1005930.s007.pdf]

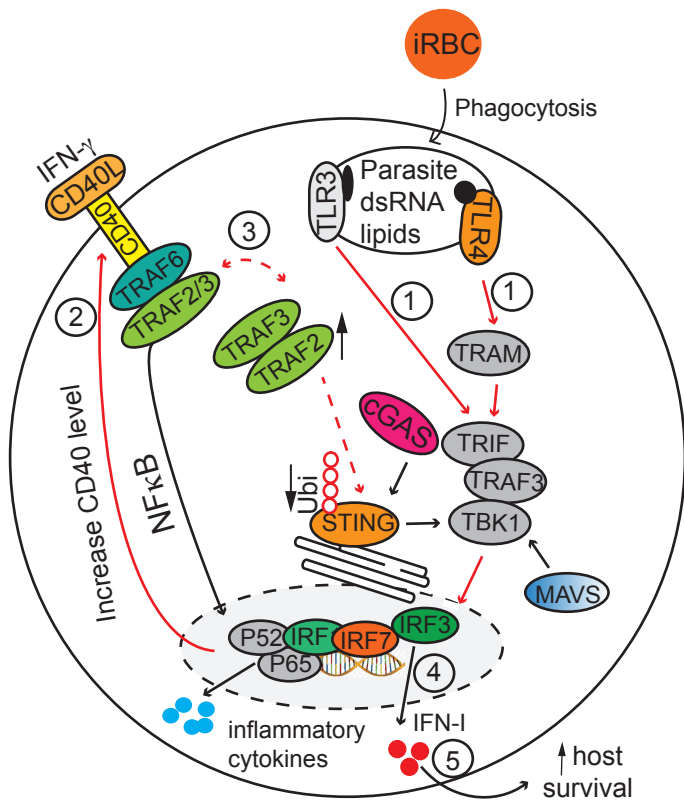

Fig. S8

Supplement: S8 Fig — First, parasite nucleic acids (or GPI/lipids) activate TLRs after phagocytosis of infected red blood cells (iRBCs), leading to activation of TRIF dependent signaling pathways. Second, TLR signaling stimulates and increases CD40 expression. Third, increased CD40 expression leads to higher STING protein level, possibly through changes on STING ubiquitination and degradation that is influenced by levels of TRAF2/3. Fourth, higher STING level greatly increases IFN-I production during early infection. Finally, higher levels of IFN-I suppresses day 4 parasitemia and enhances host survival. This CD40/STING mediated enhanced IFN-I response may also play a role in some autoimmune disorders. Note that the high level of IFN-I triggers many negative regulators that quickly suppress the production of IFN-I. (PDF) [file ppat.1005930.s008.pdf]
