# Supplementary material for: Increased CD40 Expression Enhances Early STING-Mediated Type I Interferon Response and Host Survival in a Rodent Malaria Model
Source: PLoS Pathog. 2016 Oct 7;12(10):e1005930. doi: 10.1371/journal.ppat.1005930 (PMC5055354; doi:10.1371/journal.ppat.1005930)
Supplement: S3 Fig — (A) Partial CD40 amino acid sequence with amino acid substitution sites (in red) in the TRAF binding domains and Box 2. (B) Aligned nucleotide sequences showing expected nucleotide substitutions (in red) after DNA sequencing. (C-E) Electropherograms showing the expected nucleotides (arrows) in each TRAF binding domain from Sanger dye terminator sequencing. (PDF) [file ppat.1005930.s003.pdf]

**A.** Amino acid sequence of mouse CD40 intracelular domain

VFLYIKKVVKKPKDNEILPPAARRQDPQEMEDYPGHNTAAPVQETLHGCQPVTQEDGKESRISVQERQVTDSIALRPLV

TRAF6 (Q238A; E239A) — TRAF2/3 (T255A) — Box2 (Q264A)

**B**

```
                                                                        mT6
mT23         GTCAAGAAACCAAAGGATAATGAGATCCTACCCCCTGCGGCTCGACGGCAAGATCCCCAG
mT6          GTCAAGAAACCAAAGGATAATGAGATCCTACCCCCTGCGGCTCGACGGCAAGATCCCGCG
mTb2         GTCAAGAAACCAAAGGATAATGAGATCCTACCCCCTGCGGCTCGACGGCAAGATCCCCAG
mT23/6       GTCAAGAAACCAAAGGATAATGAGATCCTACCCCCTGCGGCTCGACGGCAAGATCCCGCG
mT6/b2       GTCAAGAAACCAAAGGATAATGAGATCCTACCCCCTGCGGCTCGACGGCAAGATCCCGCG
mT23/6/b2    GTCAAGAAACCAAAGGATAATGAGATCCTACCCCCTGCGGCTCGACGGCAAGATCCCGCG
CD40WT       GTCAAGAAACCAAAGGATAATGAGATCCTACCCCCTGCGGCTCGACGGCAAGATCCCCAG
             *********************************************************  *

mT6          mT6                                          mT23
mT23         GAGATGGAAGATTATCCCGGTCATAACACCGCTGCTCCAGTGCAGGAGGCGCTGCACGGG
mT6          GCGATGGAAGATTATCCCGGTCATAACACCGCTGCTCCAGTGCAGGAGACGCTGCACGGG
mTb2         GAGATGGAAGATTATCCCGGTCATAACACCGCTGCTCCAGTGCAGGAGACGCTGCACGGG
mT23/6       GCGATGGAAGATTATCCCGGTCATAACACCGCTGCTCCAGTGCAGGAGGCGCTGCACGGG
mTb6/b2      GCGATGGAAGATTATCCCGGTCATAACACCGCTGCTCCAGTGCAGGAGACGCTGCACGGG
mT23/6/b2    GCGATGGAAGATTATCCCGGTCATAACACCGCTGCTCCAGTGCAGGAGGCGCTGCACGGG
CD40WT       GAGATGGAAGATTATCCCGGTCATAACACCGCTGCTCCAGTGCAGGAGACGCTGCACGGG
             * ********************************************** ***********

                        mTb2
mT23         TGTCAGCCTGTCACACAGGAGGATGGTAAAGAGAGTCGCATCTCAGTGCAGGAGCGGCAG
mT6          TGTCAGCCTGTCACACAGGAGGATGGTAAAGAGAGTCGCATCTCAGTGCAGGAGCGGCAG
mTb2         TGTCAGCCTGTCACAGCGGAGGATGGTAAAGAGAGTCGCATCTCAGTGCAGGAGCGGCAG
mT23/6       TGTCAGCCTGTCACACAGGAGGATGGTAAAGAGAGTCGCATCTCAGTGCAGGAGCGGCAG
mT23/b2      TGTCAGCCTGTCACAGCGGAGGATGGTAAAGAGAGTCGCATCTCAGTGCAGGAGCGGCAG
mT23/6/b2    TGTCAGCCTGTCACAGCGGAGGATGGTAAAGAGAGTCGCATCTCAGTGCAGGAGCGGCAG
CD40WT       TGTCAGCCTGTCACACAGGAGGATGGTAAAGAGAGTCGCATCTCAGTGCAGGAGCGGCAG
             ***************  ******************************************
```

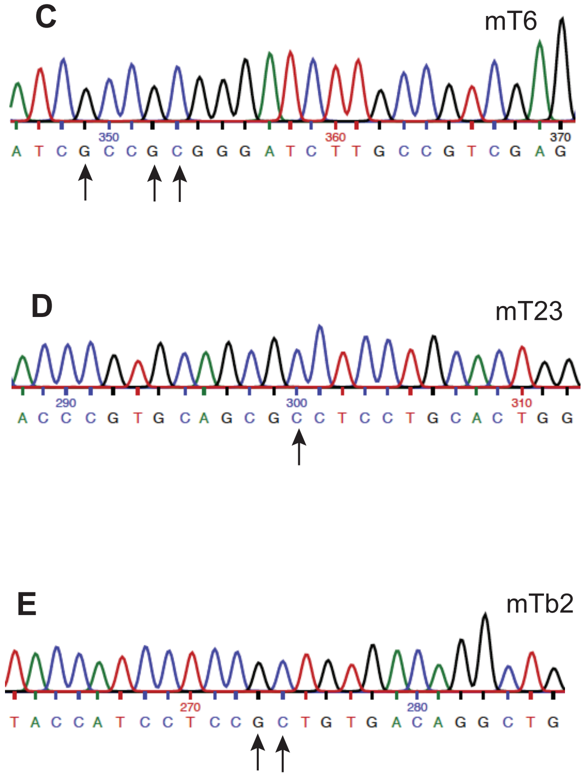


FIg S3
